# Slab to back-arc to arc: Fluid and melt pathways through the mantle wedge beneath the Lesser Antilles

**DOI:** 10.1126/sciadv.add2143

**Published:** 2023-02-01

**Authors:** Stephen P. Hicks, Lidong Bie, Catherine A. Rychert, Nicholas Harmon, Saskia Goes, Andreas Rietbrock, Songqiao Shawn Wei, Jenny S. Collier, Timothy J. Henstock, Lloyd Lynch, Julie Prytulak, Colin G. Macpherson, David Schlaphorst, Jamie J. Wilkinson, Jonathan D. Blundy, George F. Cooper, Richard G. Davy, John-Michael Kendall, VoiLA Working Group

**Affiliations:** ^1^ Department of Earth Science and Engineering, Imperial College London, London, UK.; ^2^ School of Environmental Sciences, University of East Anglia, Norwich, UK.; ^3^ School of Ocean and Earth Science, University of Southampton, Southampton, UK.; ^4^ Woods Hole Oceanographic Institution, Falmouth, MA, USA.; ^5^ Karlsruhe Institute of Technology, Karlsruhe, Germany.; ^6^ Department of Earth and Environmental Sciences, Michigan State University, East Lansing, MI, USA.; ^7^ Seismic Research Centre, The University of the West Indies, St. Augustine, Trinidad and Tobago.; ^8^ Department of Earth Sciences, Durham University, Durham, UK.; ^9^ Instituto Dom Luiz, University of Lisbon, Lisbon, Portugal.; ^10^ London Natural History Museum, London, UK.; ^11^ Department of Earth Sciences, University of Oxford, Oxford, UK.; ^12^ School of Earth and Ocean Sciences, Cardiff University, Cardiff, UK.

## Abstract

Volatiles expelled from subducted plates promote melting of the overlying warm mantle, feeding arc volcanism. However, debates continue over the factors controlling melt generation and transport, and how these determine the placement of volcanoes. To broaden our synoptic view of these fundamental mantle wedge processes, we image seismic attenuation beneath the Lesser Antilles arc, an end-member system that slowly subducts old, tectonized lithosphere. Punctuated anomalies with high ratios of bulk-to-shear attenuation (*Q*_κ_^−1^/*Q*_μ_^−1^ > 0.6) and *V_P_*/*V_S_* (>1.83) lie 40 km above the slab, representing expelled fluids that are retained in a cold boundary layer, transporting fluids toward the back-arc. The strongest attenuation (1000/*Q_S_* ~ 20), characterizing melt in warm mantle, lies beneath the back-arc, revealing how back-arc mantle feeds arc volcanoes. Melt ponds under the upper plate and percolates toward the arc along structures from earlier back-arc spreading, demonstrating how slab dehydration, upper-plate properties, past tectonics, and resulting melt pathways collectively condition volcanism.

## Teaser

Seismic attenuation maps deviant fluid and melt pathways from the subducted slab to the volcanic arc in the Lesser Antilles.

## INTRODUCTION

By delivering volatiles to the deep Earth and returning them to the surface, subduction zones are a key player in Earth’s deep water cycle. This volatile cycling generates earthquakes in the subducting slab and forms ore deposits. Volatiles also lower the solidus temperature of the mantle, which causes the mantle to melt, causing potentially hazardous eruptions along volcanic arcs (*1*–*3*). However, the fundamental controls on melt genesis and arc position at the surface remain debated, falling into two end-member hypotheses (*4*). In the first hypothesis, deep processes in the slab and mantle wedge dominate variations in magmatism, with slab devolatilization and mantle wedge thermal structure playing key roles (*5*). Alternatively, upper-plate controls such as stress state, preexisting structures (*6*), and storage are key. Understanding what dictates melt generation and transport and how these determine the location of volcanoes is vital for fully understanding hazardous subduction systems.

Subduction zone thermal structure is governed mainly by the age and velocity of the downgoing lithosphere, the background potential temperature of the mantle, as well as the depth where the slab and mantle couple mechanically (*7*, *8*). Numerical models and heat-flow data indicate a sharp coupling transition depth (hereafter CTD; also called “decoupling depth”) at ~80 km in many subduction zones (*3*, *9*). Models of mantle wedge melting typically assume that volatiles and melt rise vertically because of their positive buoyancy (*2*, *3*, *10*–*12*); slab surface temperatures inferred by some geothermometry data broadly support this (*13*). However, when considering compaction effects, some models show more complex fluid pathways through the mantle (*14*, *15*), with a likely impact on magma genesis and arc position (*16*). Melt generation and transport may also depend on variable slab hydration (*17*), properties of the thermal boundary layer (hereafter TBL; also called “viscous blanket”) atop the slab (*14*, *18*), permeability structure in the lowermost part of the upper plate (*15*, *16*, *19*–*21*), and long-term arc migration (*22*).

Strong intrinsic seismic attenuation (expressed by a high inverse quality factor, *Q*^−1^) can be caused by high temperatures and the presence of melt (*23*), thus offering a window into geodynamic processes beneath volcanic arcs. Images of *Q*^−1^ offer insights into slab dehydration (*24*), melt generation (*25*), transport mechanisms (*26*), and their relationship to volcanic output (*24*, *27*, *28*). Jointly imaging bulk and shear attenuation (*Q*_κ_^−1^ and *Q*_μ_^−1^) can help to distinguish free fluids from melt. For example, a high *Q*_κ_^−1^/*Q*_μ_^−1^ ratio (>0.8) in a low *Q*_μ_^−1^ medium occurs because of thermoelastic relaxation from fluid pockets that enhance grain-scale heterogeneity (*26*, *28*, *29*).

Previous *Q*^−1^ tomography studies have focused on Pacific-type margins that generally subduct plates, which were formed at intermediate-to-fast spreading ridges, at a relatively high rate (>4 cm/year). Tomographic images typically show a sharp lateral transition spanning less than 50 km, from low *Q*^−1^ in the rigid cold nose in the fore-arc corner to high *Q*^−1^ of the warm convecting mantle, representing the CTD (*3*, *7*). Apart from regions with active back-arc spreading, such as Tonga-Lau (*26*), the highest *Q*^−1^ lies directly beneath the volcanic front, at 50 to 100 km in depth (*24*–*28*, *30*–*32*). These *Q*^−1^ anomalies typically overlap with a region of high *V_P_*/*V_S_* (>1.8) (*33*–*35*). To first-order, these sub-arc seismic anomalies reinforce the classic paradigm once melt is generated, and it takes a mostly vertical path to the arc above. However, thermal structure and slab devolatilization depend on plate age, subduction velocity (*3*), and hydration of the incoming plate, which is influenced by the spreading rate at its formation (*17*); , existing *Q*^−1^ images do not include an important end-member of slow subduction of an old plate.

**Fig. 1.**
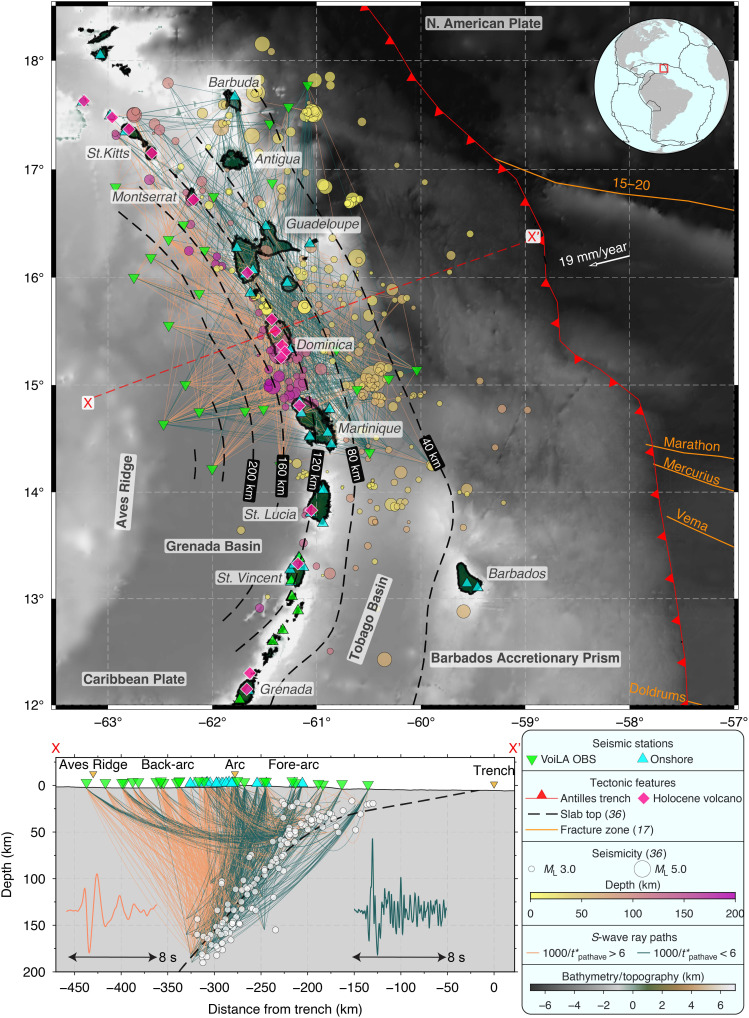
Seismotectonic context of the Lesser Antilles arc, with *S*-wave ray-path coverage and path-averaged t*_S_ results. The red box on the inset map shows the extent of the main map. Island names are labeled in italic; tectonic features are in bold. Ray-paths in the map (top) and cross-sectional view (bottom) are traced in a three-dimensional (3D) velocity model (*42*), with ray-path colors showing the path-averaged attenuation operator (t*_pathave_). Orange paths have strong attenuation; green paths have weak attenuation. The orientation of the 2D model spanning the northern LAA shown in Fig. 2 is given by the red dashed line labeled X-X′. On the cross-sectional view, representative 8-s-long *S* waveforms on thetransverse component are given for back-arc ray-paths (orange) and a fore-arc path (green) from the same intraslab earthquake at 180 km in depth (details in fig. S1).

This study therefore focuses on the end-member Lesser Antilles arc (LAA) system (Fig. 1) due to its slow consumption (~19 mm/year) of old [80 to 120 million years (Ma)], slow-spread lithosphere. The sub-arc slab depth for the north central LAA is ~120 to 140 km (*36*), deeper than the global average of 105 km (*8*, *16*), which might hint at a ~70- to 90-km-thick zone of convecting sub-arc mantle. However, the mantle is largely isotropic based on weak *S*-wave splitting (*37*). The narrow zone of arc volcanism (Fig. 1) provides an opportunity to image fundamental melt pathways through the mantle.

Past tectonics in the Eastern Caribbean may condition present-day melt pathways through the upper plate. The frontal volcanic arc on the overriding Caribbean plate stepped backward (i.e., trenchward) at 40 Ma and then forward, to its current position, at 20 Ma (*38*). Back-arc spreading accompanied these previous arcs, but there is no evidence for rifting in the back-arc Grenada Basin today (*38*), which probably arises due to minimal trench retreat in the LAA system today (*39*, *40*).

There are also lateral variations in the hydration state of the oceanic lithosphere before its subduction into the Antilles trench. Active-source seismic images reveal a heterogeneous incoming plate with alternating tectonized and magmatically robust segments (*41*). During outer rise bending at the trench, hydration is strengthened while preserving its original spatial pattern (*39*). There is also evidence for variable hydration within the subducted slab. The highest rate of intraslab, intermediate-depth earthquakes (maximum depth of 200 km) occurs in a narrow region between Martinique and Dominica (*36*), with *b* values peaking offshore of Martinique (*40*). Seismic velocities show dehydration of slab crust and serpentinized mantle at ~60 and ~150 km in depth, respectively (*42*). Serpentine-derived fluids identified via elevated levels of boron-11 isotopes (*17*) imply relatively high degrees of mantle alteration along the Marathon and Mercurius fracture zones (FZs) (Fig. 1), representing the boundary between the Proto-Caribbean and Equatorial Atlantic oceanic domains (*43*). Tomographic imaging and receiver functions (*44*–*47*) show along-arc variations in *S*-wave velocity (*V_S_*), with the slowest upper-plate mantle and asthenospheric wedge beneath Dominica, extending 100 km into the back-arc.

Crucial unanswered questions remain about the LAA. Notably, why are low *V_S_* anomalies in the back-arc mantle wedge offset from FZs, and why there is no strongly elevated *V_P_*/*V_S_* (>1.80) in the sub-arc mantle wedge (*42*, *44*) as seen beneath Pacific arcs? To address these questions, this study investigates the locations and mechanisms of flux melting in the mantle wedge and the resulting melt pathways beneath the LAA. The LAA provides a unique opportunity to examine the effects of an end-member subduction system with long-term arc migration. The largely submarine nature of ocean-ocean subduction zones presents a challenge in imaging the mantle wedge. Therefore, in this study, we use seismic data from a temporary ocean-bottom seismometer (OBS) network in the LAA (*48*) that, combined with on-island arc stations, offers robust imaging of the slab, mantle, and upper plate. We focus on the most seismically active segment of the arc, from Martinique to Montserrat (Fig. 1). We compute the whole-path attenuation operator, t*, for ~2500 *P* and *S* waves to tomographically invert for the two-dimensional (2D) and 3D variation of *Q*^−1^ (see Materials and Methods). We assume a frequency-dependent coefficient, α = 0.27, with frequencies of 1 to 6 Hz contributing to the *S*-wave spectral fitting along the most attenuating ray-paths (fig. S1). After thorough resolution tests, we interpret substantial 3D variations in *Q*^−1^. We integrate our *Q*^−1^ models with previously published seismic velocities and compare them against theoretical predictions from geodynamic models to interpret pathways of partial melts and slab-derived volatiles through the mantle wedge beneath the LAA.

## RESULTS

*P* and *S* waveforms from intermediate-depth intraslab earthquakes recorded on OBS stations in the back-arc show substantial high-frequency attenuation (fig. S1). We verify this initial result by visualizing path-averaged t* values for each ray-path (Fig. 1). In contrast to these highly attenuating ray-paths that travel up through the back-arc mantle wedge, weakly attenuating ray-paths are those that travel up through the slab and fore-arc. Within the constraints of our resolution tests and assumptions in our t* spectral fitting method, we describe a broader 2D and more detailed 3D *Q*^−1^ model for the LAA, with rays traced in a regional 3D velocity model derived from a similar earthquake dataset (*42*). Our tests show that the shape and amplitude of the main *Q*^−1^ anomalies are insensitive to assumptions about station corrections and corner frequency (see Materials and Methods). We can resolve anomalies with characteristic lengths of 25 to 50 km under the fore-arc, arc, and back-arc (see Materials and Methods for full details).

**Fig. 2.**
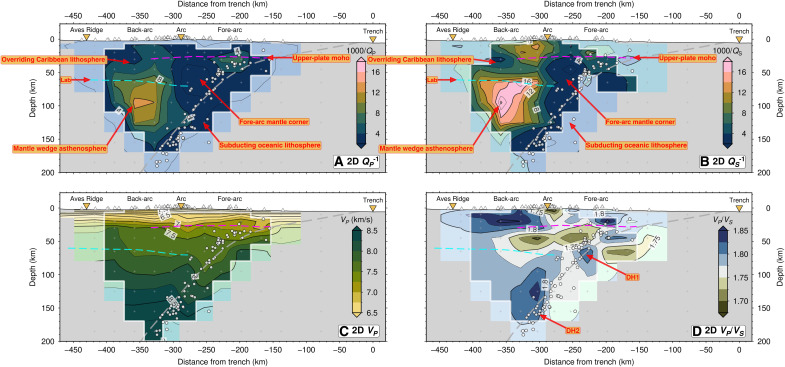
Comparison between 2-D seismic attenuation (Q^-1^) and velocity models (*42*) The thick gray dashed line is the slab interface (*36*). The magenta dashed line indicates the upper-plate Moho (*49*). The dashed cyan line indicates a negative seismic velocity discontinuity interpreted as the lithosphere-asthenosphere boundary (LAB) at the base of the Caribbean plate (*46*). White cross symbols indicate the model inversion nodes. White circles are earthquake hypocenters; white triangles are seismic stations. The cross section orientation corresponds to X-X′ shown in Fig. 1. The white line surrounding the most opaque colors denotes the resolution limit from fig. S2A. The labels “DH1” and “DH2” in (**D**) correspond to the first (slab crust) and second (slab mantle) dehydration pulses, respectively (*42*). For comparison, figure S3 shows the % change in *V_P_* relative to a 1D reference velocity model (*36*).

**Fig. 3.**
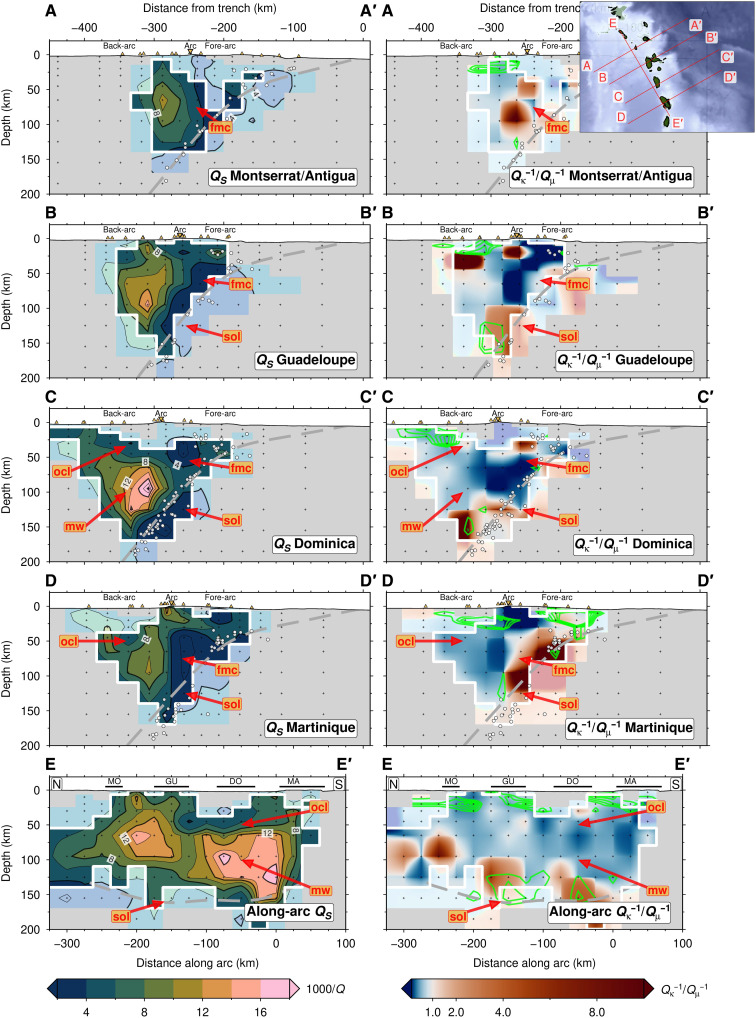
Cross sections through the 3D *Q_S_*^−1^ and *Q*_κ_^−1^/*Q*_μ_^−1^ models. The inset map (top right) shows the location of each cross-section. The top four rows (A-A′ to D-D′) are arc-perpendicular sections; the bottom row (E-E′) shows an arc-parallel section in the back-arc, with the labeled horizontal black lines showing islands (MO, Montserrat; GU, Guadeloupe; DO, Dominica; MO, Martinique). The green contours on the *Q*_κ_^−1^/*Q*_μ_^−1^ images denote zones of high *V_P_*/*V_S_* (>1.83; in intervals of 0.01) (*42*). The thick gray dashed line is the slab interface (*36*). Labeled features (fmc, fore-arc mantle corner; mw, mantle wedge; clm, Caribbean lithosphere mantle; sol, subducting oceanic lithosphere) are discussed in Results. *Q*_κ_^−1^/*Q*_μ_^−1^ is plotted with a diverging color scale to emphasize regions wher*e Q*_κ_^−**1**^ > *Q*_μ_^−1^.

**Fig. 4.**
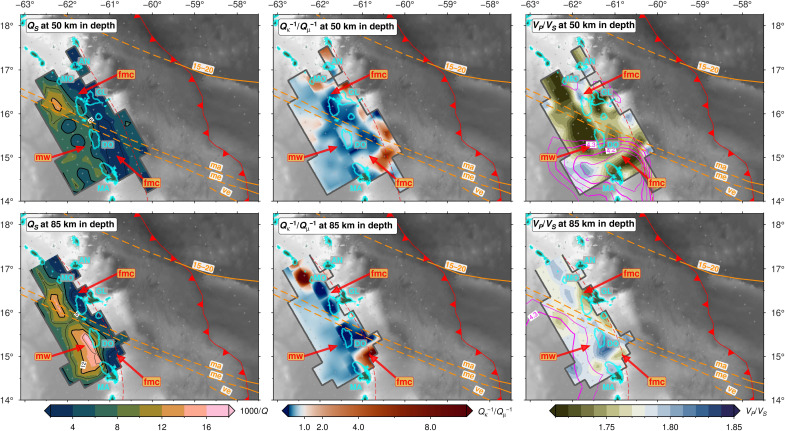
Depth sections (map view) through the 3D seismic attenuation model. Seismic properties are shown at depths of 50 km (top row) and 85 km (bottom row), with *Q_S_*^−1^ (left) and *Q*_κ_^−1^/*Q*_μ_^−1^ (middle), and *V_P_/V_S_* (*42*) and *V_S_* from teleseismic Rayleigh waves (*44*) (right). Low *V_S_* zones are highlighted by the magenta contours covering 4.15 to 4.35 km/s in intervals of 0.05 km/s. The thick cyan lines give the coastlines of islands. Fracture zones (FZs; and their projected positions) are shown as dashed orange lines (15–20, Fifteen-Twenty; ma, Marathon; me, Mercurius; ve, Vema). The slab-top at the corresponding section depth is shown by the red dashed line. Other labeled features are defined as per Fig. 3 and are discussed in Results.

Our tomographic inversions reveal considerable *Q_P_*^−1^ and *Q_S_*^−1^ variations perpendicular to and parallel to the LAA. We identify and interpret the first-order domains of the subduction zone from the 2D *Q*^−1^ inversion (Fig. 2) within the framework of structural boundaries from previous work: the upper-plate Moho (*49*), the slab top inferred from seismicity (*36*), and the upper-plate lithosphere-asthenosphere boundary (LAB) (Fig. 2) (*44*, *46*). Notably, the most prominent *Q*^−1^ anomalies do not always directly correspond to strongest *V_P_* or *V_P_*/*V_S_* anomalies, suggesting that the physical properties responsible for these different types of seismic anomalies are spatially decoupled. We present the 3D tomographic model in arc-perpendicular and depth sections in Figs. 3 and 4, respectively. Given the more substantial *S*-wave attenuation, we focus on the 3D variation of *Q_S_*^−1^ and *Q*_κ_*^−1^*/*Q*_μ_^−1^. We describe the main features of our tomographic images below.

### Subducting oceanic lithosphere (“sol”)

We find the lowest *Q*^−1^ in the subducted slab (1000/*Q* < 4), which is present across the arc and is consistent with variations in slab geometry (*36*).

### Fore-arc mantle corner (“fmc”)

Like the slab, the fore-arc mantle is weakly attenuating (1000/*Q_S_* < 4). The mantle corner appears as a large, uniformly low *Q_S_*^−1^ anomaly beneath the fore-arc and volcanic arc, extending from the upper-plate Moho at 30 km in depth to the top of the subducting plate at 120 km in depth (Fig. 2). In 3D, the low *Q_S_*^−1^ mantle corner appears persistent throughout the arc; however, its appearance varies subtly. Beneath Martinique (section D-D′), the fore-arc corner is more prominent and has a sharper, near-vertical boundary with the back-arc mantle wedge (Fig. 3). Whereas further north beneath Guadeloupe (section B-B′), the fore-arc anomaly is smaller and has a weaker contrast with the asthenospheric mantle wedge to the west. Although relatively nonattenuating, the fore-arc mantle displays an elevated *Q*_κ_^−1^/*Q*_μ_^−1^ (>0.6). In the arc-parallel profile (Fig. 3, section E-E′), this high *Q*_κ_^−1^/*Q*_μ_^−1^ anomaly has a punctuated appearance, being most prominent directly beneath the volcanic islands, especially Guadeloupe, Dominica, and Martinique.

### Mantle wedge (“mw”) asthenosphere

Below the back-arc, there is a sharp increase in *Q*^−1^ at depths greater than 60 km. We see the most prominent and highest *Q_P_*^−1^ and *Q_S_*^−1^ anomalies (1000/*Q* > 20) at depths of 60 to 140 km and, unexpectedly, 40 to 70 km west of the volcanic arc, rather than directly under the arc. We interpret this high *Q*^−1^ beneath the back-arc as the asthenospheric mantle wedge (Fig. 2). This attenuating wedge extends into the back-arc 100 km west of the volcanic arc, at least to the westernmost limit of our resolution. The high *Q*^−1^ does not seem to extend to the top of the slab, instead lying ~40 km above it. Throughout the back-arc, the high *Q_S_*^−1^ mantle wedge reaches the upper-plate LAB, where there is then a strong *Q*^−1^ gradient. The highest *Q_S_*^−1^ values in the asthenosphere wedge (1000/*Q_S_* = 17 to 25) lie at 80 to 110 km in depth beneath the back-arc of Dominica (section C-C′; Figs. 3 and 4). To the south, wedge *Q*^−1^ rapidly decreases (1000/*Q_S_* = 7 to 9) beneath Martinique (section D-D′). Compared to the fore-arc corner and the 40-km-thick layer above the slab, the core of the back-arc mantle wedge has a more moderate *Q*_κ_^−1^/*Q*_μ_^−1^ (0.4 to 0.6), similar to in the Alaska subduction zone (*28*), but less than beneath Tonga-Lau (0.75) (*26*).

### Overriding Caribbean lithosphere (“ocl”)

Our resolution tests show lateral and vertical smearing between nodes at shallow depths (<40 km). Nevertheless, we tentatively identify low *Q*^−1^ (1000/*Q_S_* = 4 to 8) sandwiched between the LAB (*46*) and Moho, with a shallower high *Q*^−1^ (1000/*Q_S_* = 8 to 12), extending from the arc to up to ~50 km west into the back-arc (Fig. 2). This anomaly may , in part, be caused by thick (up to 11 km) fluid-saturated sediments in the Grenada Basin (*38*), as evidenced by coincident high *V_P_*/*V_S_* (>1.8) (*44*). We do not have the resolution in 3D to determine how this upper-plate anomaly varies beneath the different volcanic islands (Figs. 3 and 4), and therefore, we do not interpret it further.

### Synthetic tests

**Fig. 5.**
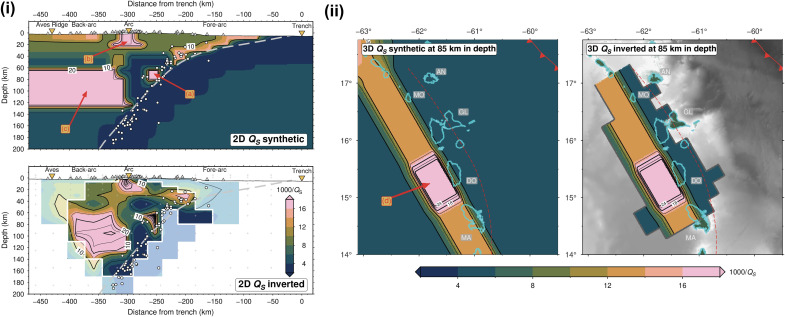
Restoring resolution tests. (i) Synthetic test for the 2D inversion showing the input model (top) and the recovery (bottom). (ii) A similar test for the 3D inversion to recover along-arc variability in mantle wedge attenuation, with the input model (left) and the recovered model (right). Alphabetically labeled features are described in Results. All labeled features are present in the tomographic models using real data (Figs. 2 and 3) apart from feature (a).

To better understand the robustness of our identified features, we designed a set of synthetic models to answer some critical questions. (a) Can we resolve a high *Q*^−1^ mantle wedge under the fore-arc that would be more consistent with a CTD of 80 km based on Pacific studies? (b) Can our inversion distinguish a high *Q*^−1^ mantle wedge from a high *Q*^−1^ in the sub-arc crust? (c) Can we successfully resolve the geometry of a high *Q*^−1^ mantle wedge beneath the back-arc and (d) image along-arc variations in its amplitude? Figure 5 shows the synthetic models with labeled anomalies corresponding to the questions above. Similar to our checkerboard tests (see Materials and Methods), we computed corresponding synthetic t* measurements, added random, normally distributed noise with an SD of 0.005 s (based on the mean SD computed the real-data t* inversions), and inverted these data, as per our actual data inversions. For these synthetic inversions, we used the 2D and 3D velocity models for the LAA from Bie *et al.* (*42*).

The resulting inversions (Fig. 5) recover the long-wavelength shapes and the absolute *Q*^−1^ values of many input anomalies. In particular, our results show that the high *Q*^−1^ anomaly in the sub-arc crust (b) is resolvable in 2D. Moreover, we can rule out the possibility of a localized high *Q*^−1^ anomaly in the fore-arc mantle wedge that would indicate a CTD at ~80 km in depth (a). We can also distinguish mantle wedge structures from high *Q*^−1^ anomalies in the upper plate (c). Last, the geometry and amplitude of the high *Q*^−1^ back-arc mantle wedge (d), with its along-arc peak near Dominica, are robust features.

## DISCUSSION

We compare the imaged seismic attenuation structure with published seismic velocity models from local earthquake tomography (*V_P_* and *V_P_*/*V_S_*) (*42*, *50*), along with *V_SV_* from teleseismic Rayleigh waves (*44*) and ambient noise (*45*). Because strong intrinsic seismic attenuation in mantle is caused by high temperatures, along with the presence of volatiles and melt, we use experimental and numerical predictions (*23*, *42*, *44*, *51*, *52*) to interpret mantle wedge thermal structure and the likely pathways of fluid and melt. We make our main interpretations in the context of the two slab dehydration pulses that are predicted from numerical models of subduction beneath the LAA (*42*, *44*) and that correspond to high *V_P_*/*V_S_* (>1.8) anomalies (*42*), indicating devolatilization of serpentinized slab crust and mantle at 60 to 80 km and >120 km in depth, respectively.

### Volatile flux beneath the fore-arc and implications for slab-mantle coupling

**Fig. 6.**
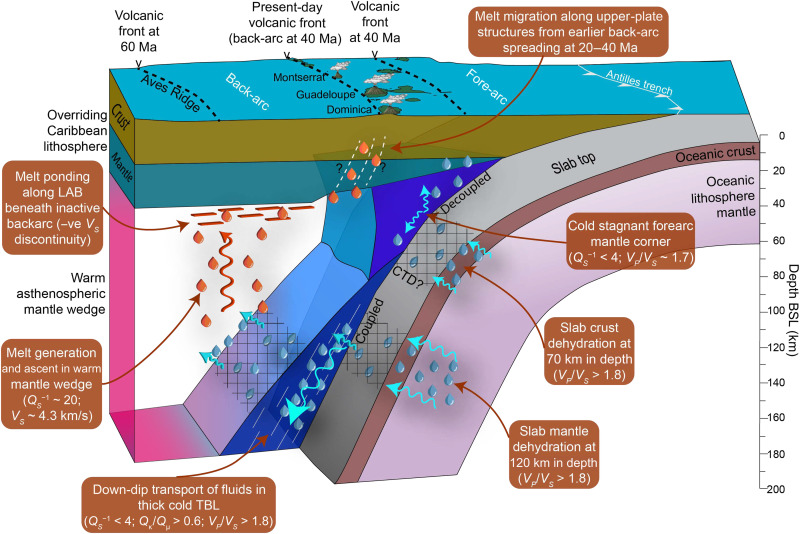
Schematic view of dehydration and melting processes in the mantle wedge beneath the Lesser Antilles Arc (LAA) based on our combined interpretation of seismic attenuation and velocities. The 3D perspective view is cut away in two locations to show the top of the slab and the top of the cold thermal boundary layer (TBL). Blue drips and arrows indicate interpreted volatiles and their pathways; red drips represent melt. The areas with hatching indicate “wet” surfaces. Approximate positions of previous volcanic arcs are plotted using the data by Allen *et al.* (*38*). Vertical exaggeration, 2× (*44*, *46*).

The fore-arc mantle that overlies the first slab dehydration peak at 60 to 80 km depth, with a *V_P_* of 7.5 to 8.2 km/s and low-moderate *V_P_/V_S_* (<1.74), is nonattenuating across the arc (1000/*Q_S_* < 4) (Figs. 2 to 4), indicating cold, melt-free mantle. There is a strong lateral gradient in *Q*^−1^ between this cold nose and the hot wedge (Figs. 2 and 3). There is no corresponding strong gradient in seismic velocity, which is influenced more by compositional changes, such as the presence of serpentine in mantle (*7*, *53*, *54*), rather than thermal variations. If we interpret the intersection of this strong lateral gradient in *Q*^−1^ with the slab top in our 2D inversion (Fig. 2), then we infer a CTD of 100 to 120 km, although its character may vary slightly along strike based on our 3D inversion (Fig. 3). A CTD of 100 to 120 km would bring its surface projection closer to the volcanic arc (*16*) but this CTD would be deeper compared to what is inferred from *Q*^−1^ images of Pacific-type subduction zones [see (*7*) and references therein]. Moreover, although seismic velocity is less sensitive to thermal structure, a deep CTD is inconsistent with the interpretation from *V_P_/V_S_* of slab crust dehydration at 60 to 80 km in depth (*42*) because the CTD controls where slab crust should fully dehydrate: normally, the blueschist-to-eclogite transition should occur within 20 km of the CTD (*15*, *42*). Regardless of the CTD beneath the LAA, the weak local *S*-wave splitting observed at stations on the island arc (~0.2 s) (*37*) supports our overall view of a large zone of cold, stagnant mantle, without vertically aligned melt, lying under the arc (Fig. 6).

Given the low-moderate *V_P_/V_S_* of the stagnant forearc mantle corner and the age of the incoming lithosphere, the expected small fluxes of these crustal-derived volatiles do not substantially serpentinize the fore-arc mantle, instead largely remaining as free fluids, similar to in other cold subduction zones (*55*). This first pulse of slab dehydration thus does not directly contribute to arc magmatism via fore-arc pathways because the mantle beneath the fore-arc and arc is too cold for sourcing the primary melts that supply the arc. Many of these fluids expelled from slab crust are likely lost in the fore-arc and facilitate the abundant seismicity in the cold mantle corner of the LAA (*36*, *50*, *56*, *57*) due to raised pore fluid pressures.

### Volatile flux and mantle wedge melting beneath the back-arc

The second peak of high *V_P_*/*V_S_* (>1.8) along the slab top lies at >140 km in depth (Fig. 2) and is interpreted as representing fluids expelled by antigorite and chlorite dehydration in the slab mantle (*42*). Our high *Q_S_*^−1^ in the back-arc mantle wedge, which extends to the upper-plate LAB at ~60 km in depth (*44*, *46*), coincides with only moderately high *V_P_*/*V_S_* (1.75 to 1.80), rather than with the highest observed *V_P_/V_S_* of 1.80 to 1.85 that lies ~10 to 20 km laterally toward the arc and ~50 km deeper in the mantle wedge (Figs. 2 to 4 and fig. S4). The *V_P_*/*V_S_* (*42*) and *Q*^−1^ inversions use the same earthquake dataset with similar imaging resolution, and we have tested the robustness of the retrieved anomalies using restoring resolution tests (Fig. 5A and fig. S5), so this offset is real and must arise from variable sensitivity of *Q*^−1^ and *V* to different material properties (*27*, *33*), which we discuss below.

In the 40-km-thick low *Q_S_*^−1^ zone atop the slab, there is, instead, some spatial overlap between high *Q*_κ_^−1^/*Q*_μ_^−1^ (>1.0) and high *V_P_*/*V_S_* (>1.83) (Fig. 3 and fig. S4). Elevated bulk attenuation may result from nonintrinsic attenuation mechanisms such as thermoelastic relaxation (*28*, *29*) or porous melt flow (*26*). However, we observe high *Q*_κ_^−1^/*Q*_μ_^−1^ in a relatively low *Q*_μ_^−1^ medium, suggesting a contribution from scattering attenuation that could be caused by isolated pockets of free fluid atop the slab that enhances grain-scale heterogeneity in cold mantle (*28*). The corresponding fast seismic velocities (*V_P_* > 8 km/s and *V_S_* > 4.45 km/s) and our *k-*means clustering analysis of seismic properties (table S1) lead us to interpret these seismic properties as being caused by a ~40-km-thick cold viscous TBL atop the slab (Fig. 6) (*1*, *18*, *58*). Numerical models predict a TBL with a high shear viscosity that allows mantle to be dragged down with the subducting plate (fig. S6), facilitating the down-dip transport of expelled slab fluids toward the back-arc (*14*). Down-dip fluid transport thus reconciles the observed offset between high *Q_S_*^−1^ and high *V_P_/V_S_* (Fig. 6).

**Fig. 7.**
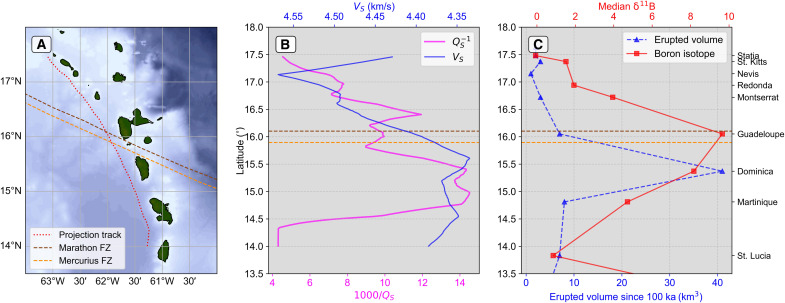
Comparison between seismic properties and magmatism along the Lesser Antilles Arc (LAA). (**A**) Map showing the line along which seismic properties are plotted in (**B**) (red dotted line) and projected FZ positions. Island labels are the same as in Fig. 4. (B) Along-arc *Q_S_*^−1^ variation at 95 km depth from this study and *V_S_* at 95 km depth (*44*). Note that the *V_S_* axis has been reversed. (**C**) Along-arc variability in total erupted volume (dashed blue line and points) (*60*) and boron isotope composition of melt inclusions from erupted volcanic rocks (red line and points) (*17*). The horizontal dashed lines in (B) and (C) show the intersection of projected subducted FZ positions (*17*) with the back-arc profile.

The highest *Q_S_*^−1^ lies in the back-arc of Dominica, correlating with low *V_S_* (~4.3 km/s) but only moderately elevated *V_P_*/*V_S_* (1.75 to 1.80) (Figs. 4 and 7B) (*42*, *44*). To understand whether this high *Q_S_*^−1^ can be explained by temperature alone, we use 2D kinematic geodynamic models [see (*44*) for methodological details] to recover the predicted thermally driven *Q_S_*^−1^ (*51*). Our models (fig. S6) predict a maximum mantle wedge temperature of (~1350°C), giving a maximum recovered 1000/*Q_S_* of only 7 to 9, which is much weaker attenuation than what we observe (1000/*Q_S_* = 17 to 25). We also tested a model of grain boundary premelting (*59*) but found that it predicts almost no attenuation (1000/*Q_S_*^−1^ ~ 0.1) for most temperatures expected in the subduction zone, only reaching a minimum 1000/*Q_S_*^−1^ of 7.5 in the core of the mantle wedge where temperatures get to within ~90% of a damp mantle solidus (see text S1 for more details). Therefore, temperature alone cannot explain the high mantle wedge attenuation.

The overlap between high *Q_S_*^−1^ and low *V_S_*, along with negligible *Q_κ_*^−1^ in the core of the mantle wedge, means that the observed anomalies likely result primarily from intrinsic rather than scattering attenuation (*25*, *32*). Moreover, seismograms from OBS stations in the back-arc, with ray-paths that traverse the attenuating wedge, show simple, low-frequency *S* waves with minimal coda (fig. S1). Therefore, assuming negligible scattering attenuation in the mantle wedge, we further investigate its properties by forward modeling *Q_S_*^−1^ and *V_S_* using the Very Broadband Rheology calculator (*52*). High *Q_S_*^−1^ in the mantle wedge cannot be explained solely by fluids because higher intrinsic attenuation trade offs with grain growth that, in turn, reduces attenuation (*23*). Having already ruled out the premelting model (*59*), we compute the likely melt fraction-temperature field using an ensemble weight of the joint probability distribution for two anelastic methods: the Andrade pseudo-period and modified Burgers models (*51*). We use the depth range of 70 to 105 km to compute averaged and conservatively representative seismic properties, accounting for standard errors (mean 1000/*Q_S_* = 16; mean *V_S_* = 4.3 km/s), from the back-arc of Dominica. Both anelastic models yield similar temperature and melt fraction distributions, and the overall ensemble result is shown in fig. S7. There is a clear trade-off between increasing temperatures and decreasing melt fractions. Still, if we take a maximum mantle wedge temperature of 1350°C from our geodynamic predictions (fig. S6), then the most likely melt fraction in the mantle wedge is 1.5 to 2.0%. A zero-melt interpretation would require unrealistically hot mantle wedge temperatures of ~1600°C (fig. S7).

Independent evidence for extensive melt comes from volcanological and geochemical constraints. Of all the volcanic islands of the LAA, Dominica, with five active volcanic centers (Fig. 1), has the highest erupted volume of magma over the last 100 ka (Fig. 7) (*60*). Moreover, Dominica-Guadeloupe is where an along-arc peak in δ^11^B values of melt inclusions indicates significant fluxing of volatiles from serpentinized slab mantle (Fig. 7) (*17*). Our *Q*^−1^ images demonstrate that these are the fluids that contribute most strongly to flux melting of the back-arc mantle.

The highest *Q*^−1^ in the back-arc mantle wedge (1000/*Q_S_* = 17 to 25) is similar to that observed in Pacific-type subduction zones where the downgoing lithosphere is consumed at a faster rate, such as Nicaragua (*27*), Marianas (*25*), New Zealand (*24*), and Tonga-Lau (*26*). In most of these subduction zones, there is typically a broad zone of high attenuation (1000/*Q_P_* > 10; 1000/*Q_S_* > 12) in the mantle wedge directly beneath the volcanic front (*27*, *31*, *32*, *61*, *62*). The exception to this pattern is Tonga-Lau, where sub-arc attenuation is low, and back-arc attenuation is high, which is similar to our result of the LAA (Fig. 2) with slow *V_S_* (<4.3 km/s) extending some 200 km into the LAA back-arc (*44*). However, for Tonga-Lau, this attenuation pattern is instead likely related to active back-arc spreading and hence decompression melting (*26*). Our result is thus counterintuitive in that, in contrast to the Lau Basin, there is no evidence of active spreading today in the Grenada Basin behind the LAA (*38*). A key implication, therefore, is that melts in the mantle wedge, which are sourced from slab mantle derived volatiles, eventually reach the active volcanic arc by taking an indirect, nonvertical pathway (Fig. 6). With high *Q*^−1^ and low *V_S_* (*42*, *44*) extending up to the base of the overriding Caribbean plate and offset from the active arc (fig. S9C), where there is a coincident negative *V_S_* gradient (*44*, *46*), we favor a model of ponding of partial melt along the LAB (*63*) beneath the back-arc (Fig. 6).

In the along-arc direction (Fig. 3, section E-E′), the highest *Q_S_*^−1^ in the mantle wedge lies atop high *Q*_κ_^−1^/*Q*_μ_^−1^ and high *V_P_/V_S_* in the TBL, suggesting a direct link between mantle wedge melting and preexisting slab hydration. However, the highest *Q_S_*^−1^ anomaly in the mantle wedge near Dominica does not spatially coincide with any projected positions of subducted hydrated FZs (*19*, *44*), with the Marathon and Mercurius FZs projected ~100 km to the north-northwest (Figs. 4 and 7B). We attribute this offset to a geometric effect that results from the oblique subduction of FZs combined with the down-dip transportation of fluids in the TBL and subsequent migration of melt from the back-arc to the arc in the opposite direction to plate convergence.

### Implications for arc volcanism

Our result offers a model that explains volatile pathways and melting from slab to arc (Fig. 6). Expelled volatiles from the slab crust dehydration (first dehydration pulse) do not likely enter the warm asthenospheric wedge and thus do not contribute substantially to flux melting in the mantle because of the overlying large cold forearc corner. However, we cannot exclude the possibility that the TBL transports small amounts of these crustal-derived fluids down-dip (*14*). Volatiles from the second, deeper pulse of slab dehydration are carried further down-dip in the cool, viscous TBL atop the slab. These fluids are eventually released into the back-arc mantle, resulting in the generation of melts beneath the back-arc (*64*) that are transported upward to the LAB of the overriding plate. The lack of active back-arc spreading (*38*), along with a negative *V_s_* gradient with depth (to <4.4 km/s) (*44*, *46*) at 60 km beneath the back-arc, indicates melt ponding at the base of the mostly cool upper plate (Fig. 6).

Previously, mechanisms of melt ponding beneath the upper plate in a subduction zone setting have been associated with gaps in arc volcanism (*21*). However, the seismic attenuation and velocity structure of the LAA (*42*, *44*, *46*) imply that most generation of melt and its subsequent ponding happen beneath the back-arc of the Dominica segment, the most magmatically productive island of the entire LAA in recent times (Fig. 7). Given that accumulated melt at the LAB must reach the active volcanoes, an outstanding question is as follows: What controls the localization of the frontal arc? We suggest that past tectonic history is a key factor here: the LAA migrated trenchward at 40 Ma from the Aves Ridge to the Limestone Caribees, followed by a forward step to its present-day position at 20 Ma, which was the previous back-arc spreading axis at 20 to 40 Ma (Fig. 6). Back-arc spreading accompanied arc volcanism at these two earlier phases (*38*). Thus the forward jump at 20 Ma built the present-day volcanic front along the preceding back-arc spreading center (Fig. 6). Therefore, today, melt is channeled and focused (*16*) from the back-arc to the arc along a permeability boundary with inclined decompaction channels along the LAB (*15*, *16*), migrating toward a pinch zone with thinner and more permeable sub-arc lithosphere left by the previous back-arc spreading center (*14*, *15*, *20*, *21*). Receiver functions verify this model by highlighting abnormally thin sub-arc lithosphere (40 km) beneath Dominica (*47*). Melt migration is further facilitated by arc-normal tension (*6*), consistent with observed tectonic structures along the LAA (*65*). Melt channels through the upper plate are likely very narrow (e.g., ~15 km in width) (*5*, *19*) and hence not imageable with our methodology. A further question remains over why melts are prevented from ascending vertically through the upper plate into the back-arc. Permeability may be reduced by the low temperature of the upper plate beneath the back-arc (*16*), as supported by seismic velocities (*44*), therefore promoting crystallization (*21*). Overall, our model uniquely involves simultaneous ponding and volcanism (Fig. 6), previously thought to individually represent end-member steady-state subduction and slab advance configurations (*21*).

Therefore, the classic paradigm in which volatiles and associated melts travel vertically from the slab to sub-arc crustal magma chambers is not universally true. Instead, we have shown that although volatiles can be released from the sub-arc slab, fluid and melt trajectory can be more circuitous, with the back-arc mantle, rather than the sub-arc wedge, acting as the main reservoir of arc magma. Geodynamic models that include compaction mechanisms predict a similar trajectory (*14*, *15*). The following critical conditions make this melt trajectory particularly extreme in the LAA: (i) subduction of old lithosphere, which causes deep dehydration of the slab mantle; (ii) slow plate convergence and, hence, low slab sinking velocity that generates a thick, high shear viscosity, and cold TBL with weak grain growth (and hence small grain size) that promotes down-dip transportation of fluids toward the back-arc (*14*, *64*); and (iii) historical migration of the arc and upper plate that preconditions its permeability structure. Yet down-dip fluid migration may still occur in thinner TBLs atop younger slabs, transporting fluids expelled at shallower depths (*14*). Moreover, arc migration is common in many subduction zones (*22*). Therefore, our observations for the LAA represent an end-member case that makes lateral fluid and melt pathways more apparent, but there may be more subtle evidence of these processes in other subduction zones. These subtle effects might be apparent in published *Q*^−1^ tomography results; a revaluation of these might be required in light of our results.

Overall, our result for the LAA demonstrates how feedback between processes across the entire subduction system, such as slab dehydration, melt pathways in the mantle, and tectonic evolution of both the subducting and upper plates, governs arc magmatism. Our melt ponding model has implications for arc productivity, whether melt supply to the arc is steady state or episodic, and how the LAA will further evolve in the future. Future petrological and geochemical studies should assess whether there is a signature in LAA lavas of magmatic reequilibration due to melt ponding at the LAB.

We have studied the seismic attenuation structure of a global end-member subduction zone in the Eastern Caribbean and integrated our results with previously determined seismic velocities. A large, weakly attenuating, and hence cold, mantle corner beneath the fore-arc and volcanic arc shows that melts cannot ascend along a vertical path from slab to arc. High bulk-to-shear attenuation (*Q*_κ_^−1^/*Q*_μ_^−1^ > 0.6) and high *V_P_/V_S_* (> 1.83) in a 40-km-thick layer above the slab reveal a cold TBL that facilitates down-dip transport of fluids at the base of the mantle wedge. Fluids being transported by the TBL before being released into the warm convecting wedge could affect estimates of slab surface temperatures from geochemical markers. Once removed from the TBL, the fluids ascend into the hot mantle wedge beneath the back-arc, where substantial melt fractions (1 to 2%) explain high *Q_S_*^−1^ (1000/*Q_S_* = 17 to 25). Interpreting seismic properties in the context of the past tectonic history in the Eastern Caribbean highlights feedback mechanisms between slab dehydration, mantle wedge melt transport, and the long-term tectonic evolution of the subduction system. We infer that melt accumulates at the base of the overriding plate below the back-arc. Some of this melt reaches the arc via an inclined pathway along the LAB. It then percolates through the upper plate via extensional structures formed previously during back-arc spreading before the arc jumped forward to its current position at 20 Ma. Fluid transport toward the back-arc in the cold TBL explains why interpreted zones of melt are spatially offset from areas of enhanced plate hydration along subducting FZs and associated domain boundary. Our study has allowed us to differentiate free fluids from melt in the mantle wedge, highlighting a subvertical pathway conditioned by a combination of mantle wedge conditions and structures inherited from the tectonic history of the arc. These signatures are made more evident by the slow subduction of old, tectonized lithosphere beneath the LAA, enhancing deep dehydration and causing a thicker TBL than Pacific-type subduction zones. Even if not as easy to image, similar feedback processes will likely govern melt supply to the volcanic arc in other subduction zones.

## MATERIALS AND METHODS

### Seismic data collection and preprocessing

Our data come from the VoiLA (Volatiles in the Lesser Antilles) experiment, which included an OBS deployment from March 2016 to May 2017 (*36*, *48*) International Federation of Digital Seismograph Networks (FDSN) network code: XZ (2016). The 34-station OBS network (Fig. 1) significantly extends the coverage of existing permanent seismic networks on the island arc, improving the resolution capability in the fore-arc and back-arc. We included stations from existing land networks in our study, with the corresponding FDSN network codes as follows: G (*66*), GL, MQ, TR, and WI (*67*).

Our local earthquake catalog (Fig. 1) (*36*) includes arrival times, local magnitudes (*M*_L_), and relocations inside a region-specific 1D velocity model from the VoiLA OBS network and existing land stations. To eliminate possible complexities in ray-path propagation effects for shallow paths (*25*, *68*) and poorly constrained hypocentral locations at shallow depths, we only used events with a hypocentral depth of greater than 15 km. We excluded events with poor location constraints, filtering with a maximum azimuthal gap of 220°. Our starting catalog has 296 events with these criteria, with magnitudes ranging from *M*_L_ 2.0 to 6.6. Before the t* inversion, we corrected the seismograms for instrument response, converted them to displacement, and rotated the horizontal components into a radial-transverse coordinate system.

### Inversion for t*

We inverted amplitude spectra of *P* and *S* waves for the path-averaged attenuation operator, t*. We followed a similar strategy to Wei and Wiens (*26*), which follows the broad inversion approach taken in several previous attenuation tomography studies in subduction zones (*25*–*28*, *68*). This consistent approach allows us to more robustly compare imaged *Q*^−1^ values from the LAA with other subduction zones.

We inverted amplitude spectra of *P* and *S* waves for each event-station pair for the attenuation operator, t*. For the *k*-th earthquake recorded at the *j*-th station, the displacement spectrum is defined as

$$A_{jk}(f_{i})=\frac{C_{jk}M_{ok}e^{-\pi f_{i}^{1-\alpha }t_{0jk}^{*}}}{1+{\left(\frac{f_{i}}{f_{ck}}\right)}^{2}}$$ (1)

where *C_jk_* is a constant factor for each observation accounting for geometric spreading, the free surface effect and source radiation (*69*); *M_0k_* and *f_ck_* are the seismic moment and corner frequency, respectively; t*_0*jk*_ is the attenuation operator at 1 Hz; and α expresses the frequency dependence of attenuation (*70*). We used a 1D velocity model for the LAA (*36*) for computing the *C_jk_* corrections. We used a nonnegative least-squares inversion to solve for t*_0*jk*_, and *M_0k_* and *f_ck_* for each event.

For each earthquake, we first computed the best-fitting corner frequency and moment using a grid search within a range of prescribed stress drops, Δσ, varying from 0.1 to 100 MPa (*25*), which is within typical observed Δσ values (*36*), assuming circular rupture and a given empirical relationship between *M*_L_ and moment magnitude (*M*_w_)

$$f_{c}=0.49\beta {\left(\frac{\text{Δ}\sigma }{M_{0}}\right)}^{\frac{1}{3}}$$ (2)

where β is the *S*-wave velocity at the hypocenter source depth (*36*). We computed *M*_0_ from a regression between *M*_L_ and *M*_w_ calculated from waveform moment tensor inversion of the VoiLA dataset (*71*)

$$M_{w}=1.05\;M_{L}-0.42$$ (3)

The resulting spectral-derived *M*_w_ values from *P* and *S* waves are consistent and are similar to corresponding *M*_L_ values (fig. S8) (*36*), showing that our inversions recover reasonable source parameters.

We selected appropriate window lengths for computing spectra. We found that 3-s-long windows, starting 0.5 and 1.0 s before the manually picked arrival for *P* and *S* waves, respectively, produced the greatest number of good-fitting t* observations (fig. S9). Longer windows introduced a bias due to secondary phases. We computed signal and noise spectra using a multitaper approach (*72*). A t* measurement was acceptable if it had a spectral misfit of <20%. Figure S1 shows an example of the t* fitting process for an example event at 182 km and recorded at stations situated in the back-arc, arc, and fore-arc. We used the vertical component for *P* waves and found the widest bandwidth where the signal-to-noise ratio exceeds 2.0, with a minimum frequency bandwidth of 2 Hz, to determine the frequency range used for the t* inversion. We used the transverse component for *S* waves, ensuring a minimum signal-to-noise ratio of 1.8 and a minimum frequency bandwidth of 1.2 Hz. The transverse component minimizes the effect of potential *P*-to-*S* conversions (*28*). We excluded frequencies below 0.5 Hz for both *P* and *S* waves to avoid ocean swell noise.

Inverting for t* requires assumptions about the remaining parameters of Eq. 1, *f_c_* and α. We experimented with different assumptions about *f_c_*. First, we required that the best-fitting *f_c_* lies within the frequency band of spectral fitting (Fig. 2B). This approach avoids unrealistic values of corner frequency in the t* inversion due to inherent trade-offs between the *f_c_* source term and the t* path term. At least four high-quality spectral observations were required to determine *f_c_* for an event. Although *f_c_* and *M_0_* can be computed separately for *P* and *S* waves, the latter on OBS records are often band-limited, resulting in a poorly constrained *f_c_*, which results in fewer *S*-wave t* observations. Therefore, alternatively, we could require that *f_c_* for *S* waves is equal to that of *P* waves (*26*) or that they differ by a scaling factor of 1.5 as theoretically expected for circular ruptures (*25*, *73*). We chose the assumptions for our dataset that produced the greatest number of good-fitting t* measurements. Our resulting preference was to assume *f*_*c*(*S*) *=*_ *f*_*c*(*P*)_ (*26*). Even with this assumption, moment magnitudes from *S*-wave spectra closely follow those from *P* waves (fig. S8). We also experimented with varying the frequency-dependent term, α. We found that, when α exceeds 0.6, the computed *M*_w_ deviated from *M*_L_, yielding unrealistic magnitudes. We found a weakly constrained minimum in *P*-wave spectral misfits at α = 0.30 if we included the deepest events in the dataset (>175 km in depth), which will have the longest paths through the mantle wedge. We used α = 0.27 because it is consistent with experimental results relevant to the mantle wedge (*51*, *53*, *74*), and so our results can be directly compared with published attenuation studies of other subduction zones (*25*, *27*, *28*, *31*). Although frequency dependence affects individual t* values, it is unlikely to affect overall *Q*^−1^ patterns in the final tomographic images (*75*).

Because the main aim of our study is to analyze mantle structure in the LAA, we considered possible frequency-dependent site effects caused by shallow crustal geological heterogeneity. Instead of inverting for a constant t* station term in the tomographic inversion, we estimated residual spectra (*28*, *68*). We stacked and smoothed residual spectra for each station and assigned the resulting median spectrum to the site effect. Site spectra (figs. S10 and S11) show no systematic site effects reflecting the local geology and the station’s position in the subduction zone (i.e., back-arc versus arc versus fore-arc). We then repeated the t* inversion process after removing the site effects from the original spectrum. Removal of the site effects reduced spectral misfit by correcting for spectral peaks and holes. This process allowed 14 and 40% more *P*- and *S*-wave t* observations, respectively, to be used. However, the final *Q*^−1^ inversions do not substantially change when removing the site effects (fig. S12).

With our optimum assumptions described above, we are left with a database of 2245 and 1557 good-fitting t* observations from 135 events for *P* and *S* waves, respectively (table S1). For weakly attenuating paths, we typically fit *P*-wave spectra up to 20 Hz on OBS stations, strongly attenuating ray-paths limit *S*-wave bandwidths to <6 Hz (fig. S1). Comparing t* for *P* and *S* waves for the same event-station paths indicates an overall *Q_P_*/*Q_S_* ratio of ~1.5. We did not find any obvious spatial pattern in path-averaged *Q_P_/Q_S_*.

### Attenuation imaging method

We restrict the areal extent of tomographic imaging by only including events and stations within the region of dense ray-path coverage along the linear arc segment from St. Kitts in the north to Saint Lucia in the south (Fig. 1). This refined area leaves a dataset of 122 events, with 1499 *P*-wave observations and 1039 *S*-wave observations. We inverted t* measurements for *Q*^−1^ images using iterative damped least-squares (*76*) and ray tracing based on a 3D seismic velocity model for the LAA developed using travel times from the same local earthquake dataset (*42*). We weight each t* observation relative to the computed spectral misfit. We determined the damping parameter for each inversion by evaluating trade-off curves between data and model parameter variance. For the tomographic inversions, the homogeneous *Q*^−1^ starting model came from the path-averaged t* for *P* and *S* waves individually (1000/*Q_P_* = 1.6 and 1000/*Q_S_* = 4.3). We also jointly inverted for bulk and shear moduli attenuation (*Q*_κ_^−1^ and *Q*_μ_^−1^, respectively) using *P*- and *S*-wave t* data for the same source-receiver pair to compute a *Q*_κ_^−1^/*Q*_μ_^−1^ ratio (*28*). We used 505 *P*- and *S*-wave observation pairs for this joint inversion.

Our first aim was to determine the arc-perpendicular structure of the subduction zone before looking into possible along-arc variations. Therefore, we generated a 2D inversion grid aligned perpendicular to the arc and trench. The grid was identical to that used by Bie *et al.* (*42*) to perform velocity tomography from the same local earthquake dataset. The spatial variation of ray-path derivative weight sum (DWS) guided the grid design. In the horizontal direction, there is a minimum grid spacing of 25 km in the model’s center, beneath the inner fore-arc, arc, and eastern back-arc, where there is the highest ray density. There is a vertical spacing of 10 km between 0 and 30 km in depth in the upper-plate crust, increasing to 20 km between 45 and 65 km in depth, and a 30 km in depth spacing between 65 and 200 km in depth in the mantle wedge region (fig. S2). For the 2D inversion, rays in 3D are traced in a 2D seismic velocity model and attenuation is inverted on a 3D grid of nodes. For the 3D tomographic imaging, we used a grid spacing of 25 km in the arc-parallel direction. Compared to the 2D inversion, the 3D model reduces overall data variance for the same t* dataset by 40 and 26% for *P* and *S* waves, respectively, which are statistically significant to within the 95% confidence level, based on *f*-test analyses that are computed in the simul2000 tomography code.

### Assessment of model resolution

We assessed model resolution based on several analyses (fig. S2) (*77*). We evaluated the diagonal element of the model resolution matrix, the spread function, and the 70% contour of each row of the resolution matrix. The results are shown for the 2D inversion in fig. S2A and the 3D inversion in figs. S13 and S14, respectively. For the *Q*_κ_^−1^/*Q*_μ_^−1^ image, we took the resolution limit from the 3D *Q_S_* inversion. We also carried out recovery tests using checkerboards in which we designed anomaly patterns on the basis of our inversion grid (whose spacing is nonuniform) with two grid configurations: (i) a coarse (2 × 2 grid spacing; i.e., a minimum 50 km–by–50 km anomalies in the center of the model) (Fig. 4Bi) and (ii) fine (1 × 1 grid spacing; i.e., a minimum 25 km–by–25 km grid spacing in the center of the model) (Fig. 4Bii). We based checkerboard amplitudes on the low *Q*^−1^ from the tomographic starting model and a high *Q*^−1^ of 1000/Q = 50. The results for the checkerboard tests with the 3D inversion are shown in figs. S15 and S16.

These tests show that we can resolve the top of the downgoing plate from ~140 km inboard of the trench to ~160 km in depth, close to the deepest seismicity beneath the LAA. Most smearing occurs in the vertical direction or toward the back-arc at shallower depths. We can image the supraslab area in the back-arc to 130 km west of the arc and in the fore-arc to ~100 km east of the arc. Resolution is best in the mantle wedge region between 40 and 140 km in depth, where the spread function is low (<2), and smearing contours indicate minimal smearing in the vertical direction (fig. S2A). We use a corresponding spread function value to indicate the region with little smearing, which we show as the region of good resolution in the tomographic images delineated by a thick white line. For the 2D inversion, we consistently resolve the structure of the 50 km–by–50 km anomalies in the mantle wedge and fore-arc and recover their *Q* amplitudes to within ~8% of the input in the mantle wedge region (fig. S2Bi). We are also able to resolve the alternating patterns of 25 km–by–25 km anomalies, although resolution diminishes in the back-arc and at shallow depths (<20 km) (fig. S2Bii). The amplitudes of the high *Q*^−1^ anomalies are also muted (~20% recovery in the mantle wedge region) with the finer-scale checker-pattern anomalies. For the 3D inversion (figs. S7 and S8), we cannot resolve the upper plate at crustal depths beneath Dominica due to the lack of broadband stations on the island. In contrast, at mantle wedge depths, the resolution is strongest in the Dominica region due to the high rate of intermediate-depth seismicity in this region of the LAA. There is more smearing in the Montserrat-Guadeloupe region due to the lack of deep seismicity. The 3D checkerboard tests (figs. S9 and S10) show diminished resolution, and we cannot consistently resolve anomalies with dimensions of <50 km.

### Testing assumptions of the t* inversion on the tomographic results

We have assumed that *f*_*c*(*S*)_ = *f*_*c*(*P*)_, although other studies use *f*_*c*(*S*)_ = *f*_*c*(*P*)_/1.5 (*25*, *73*). We have also removed site spectra before taking t* measurements. It is worth considering whether these assumptions introduce potential biases into our tomographic inversions. Therefore, we carried out two additional 2D inversions of *Q_S_*^−1^, accounting for each of these assumptions individually. The results are shown in fig. S12. These inversions are consistent with the main anomaly shapes and amplitudes as per our main inversion result.

## Supplementary Materials

### This PDF file includes:

Text S1 Table S1 Figs. S1 to S19 References

## References

[1] R. J. Stern, Subduction Zones. Rev. Geophys. 40, 3-1–3–38 (2002).

[2] E. M. Syracuse, P. E. van Keken, G. A. Abers, The global range of subduction zone thermal models. Phys. Earth Planet. Inter. 183, 73–90 (2010).

[3] I. Wada, K. Wang, Common depth of slab-mantle decoupling: Reconciling diversity and uniformity of subduction zones. Geochem. Geophys. Geosyst. 10, Q10009 (2009).

[4] C. B. Till, A. J. R. Kent, G. A. Abers, H. A. Janiszewski, J. B. Gaherty, B. W. Pitcher, The causes of spatiotemporal variations in erupted fluxes and compositions along a volcanic arc. Nat. Commun. 10, 1350 (2019). 30902993 10.1038/s41467-019-09113-0 PMC6430768

[5] A. Perrin, S. Goes, J. Prytulak, D. R. Davies, C. Wilson, S. Kramer, Reconciling mantle wedge thermal structure with arc lava thermobarometric determinations in oceanic subduction zones. Geochem. Geophys. Geosyst. 17, 4105–4127 (2016).

[6] H. Andikagumi, C. G. Macpherson, K. J. W. McCaffrey, Upper plate stress controls the distribution of Mariana arc volcanoes. J. Geophys. Res. Solid Earth 125, e2019JB017391 (2020).

[7] G. A. Abers, P. E. van Keken, C. R. Wilson, Deep decoupling in subduction zones: Observations and temperature limits. Geosphere 16, 1408–1424 (2020).

[8] E. M. Syracuse, G. A. Abers, Global compilation of variations in slab depth beneath arc volcanoes and implications. Geochem. Geophys. Geosyst. 7, Q05017 (2006).

[9] B. C. Kerswell, M. J. Kohn, T. V. Gerya, Backarc lithospheric thickness and serpentine stability control slab-mantle coupling depths in subduction zones. Geochem. Geophys. Geosyst. 22, e2020GC009304 (2021).

[10] N. Harmon, D. K. Blackman, Effects of plate boundary geometry and kinematics on mantle melting beneath the back-arc spreading centers along the Lau Basin. Earth Planet. Sci. Lett. 298, 334–346 (2010).

[11] H. Iwamori, Transportation of H_2_O and melting in subduction zones. Earth Planet. Sci. Lett. 160, 65–80 (1998).

[12] J. H. Davies, D. J. Stevenson, Physical model of source region of subduction zone volcanics. J. Geophys. Res. Solid Earth 97, 2037–2070 (1992).

[13] L. B. Cooper, D. M. Ruscitto, T. Plank, P. J. Wallace, E. M. Syracuse, C. E. Manning, Global variations in H_2_O/Ce: 1. Slab surface temperatures beneath volcanic arcs. Geochem. Geophys. Geosyst. 13, Q03024 (2012).

[14] N. G. Cerpa, I. Wada, C. R. Wilson, Fluid migration in the mantle wedge: Influence of mineral grain size and mantle compaction. J. Geophys. Res. Solid Earth 122, 6247–6268 (2017).

[15] C. R. Wilson, M. Spiegelman, P. E. van Keken, B. R. Hacker, Fluid flow in subduction zones: The role of solid rheology and compaction pressure. Earth Planet. Sci. Lett. 401, 261–274 (2014).

[16] G. Ha, L. G. J. Montési, W. Zhu, Melt focusing along permeability barriers at subduction zones and the location of volcanic arcs. Geochem. Geophys. Geosyst. 21, e2020GC009253 (2020).

[17] G. F. Cooper, C. G. Macpherson, J. D. Blundy, B. Maunder, R. W. Allen, S. Goes, J. S. Collier, L. Bie, N. Harmon, S. P. Hicks, A. A. Iveson, J. Prytulak, A. Rietbrock, C. A. Rychert, J. P. Davidson; VoiLA team, Variable water input controls evolution of the Lesser Antilles volcanic arc. Nature 582, 525–529 (2020). 32581382 10.1038/s41586-020-2407-5

[18] C. Kincaid, I. S. Sacks, Thermal and dynamical evolution of the upper mantle in subduction zones. J. Geophys. Res. Solid Earth 102, 12295–12315 (1997).

[19] P. C. England, R. F. Katz, Melting above the anhydrous solidus controls the location of volcanic arcs. Nature 467, 700–703 (2010). 20930842 10.1038/nature09417

[20] A.-M. Cagnioncle, E. M. Parmentier, L. T. Elkins-Tanton, Effect of solid flow above a subducting slab on water distribution and melting at convergent plate boundaries. J. Geophys. Res. Solid Earth 112, B09402 (2007).

[21] S. Rondenay, L. G. J. Montési, G. A. Abers, New geophysical insight into the origin of the Denali volcanic gap. Geophys. J. Int. 182, 613–630 (2010).

[22] B. R. Jicha, S. M. Kay, Quantifying arc migration and the role of forearc subduction erosion in the central Aleutians. J. Volcanol. Geotherm. Res. 360, 84–99 (2018).

[23] G. A. Abers, K. M. Fischer, G. Hirth, D. A. Wiens, T. Plank, B. K. Holtzman, C. McCarthy, E. Gazel, Reconciling mantle attenuation-temperature relationships from seismology, petrology, and laboratory measurements. Geochem. Geophys. Geosyst. 15, 3521–3542 (2014).

[24] D. Eberhart-Phillips, S. Bannister, M. Reyners, Attenuation in the mantle wedge beneath super-volcanoes of the Taupo Volcanic Zone, New Zealand. Geophys. J. Int. 220, 703–723 (2020).

[25] S. H. Pozgay, D. A. Wiens, J. A. Conder, H. Shiobara, H. Sugioka, Seismic attenuation tomography of the Mariana subduction system: Implications for thermal structure, volatile distribution, and slow spreading dynamics. Geochem. Geophys. Geosyst. 10, Q04X05 (2009).

[26] S. S. Wei, D. A. Wiens, High bulk and shear attenuation due to partial melt in the Tonga-Lau back-arc mantle. J. Geophys. Res. Solid Earth 125, e2019JB017527 (2020).

[27] C. A. Rychert, K. M. Fischer, G. A. Abers, T. Plank, E. Syracuse, J. M. Protti, V. Gonzalez, W. Strauch, Strong along-arc variations in attenuation in the mantle wedge beneath Costa Rica and Nicaragua. Geochem. Geophys. Geosyst. 9, Q10S10 (2008).

[28] J. C. Stachnik, G. A. Abers, D. H. Christensen, Seismic attenuation and mantle wedge temperatures in the Alaska subduction zone. J. Geophys. Res. Solid Earth 109, B10304 (2004).

[29] B. Budiansky, E. E. Sumner Jr., R. J. O’Connell, Bulk thermoelastic attenuation of composite materials. J. Geophys. Res. Solid Earth 88, 10343–10348 (1983).

[30] X. Liu, D. Zhao, S. Li, Seismic attenuation tomography of the Northeast Japan arc: Insight into the 2011 Tohoku earthquake (Mw 9.0) and subduction dynamics. J. Geophys. Res. Solid Earth 119, 1094–1118 (2014).

[31] J. Nakajima, S. Hada, E. Hayami, N. Uchida, A. Hasegawa, S. Yoshioka, T. Matsuzawa, N. Umino, Seismic attenuation beneath northeastern Japan: Constraints on mantle dynamics and arc magmatism. J. Geophys. Res. Solid Earth 118, 5838–5855 (2013).

[32] B. Schurr, G. Asch, A. Rietbrock, R. Trumbull, C. Haberland, Complex patterns of fluid and melt transport in the central Andean subduction zone revealed by attenuation tomography. Earth Planet. Sci. Lett. 215, 105–119 (2003).

[33] E. M. Syracuse, G. A. Abers, K. Fischer, L. MacKenzie, C. Rychert, M. Protti, V. González, W. Strauch, Seismic tomography and earthquake locations in the Nicaraguan and Costa Rican upper mantle. Geochem. Geophys. Geosyst. 9, Q07S08 (2008).

[34] M. Reyners, D. Eberhart-Phillips, G. Stuart, Y. Nishimura, Imaging subduction from the trench to 300 km depth beneath the central North Island, New Zealand, with *Vp* and *V*p/*V*s. Geophys. J. Int. 165, 565–583 (2006).

[35] J. A. Conder, D. A. Wiens, Seismic structure beneath the Tonga arc and Lau back-arc basin determined from joint *Vp*, *Vp*/*Vs* tomography. Geochem. Geophys. Geosyst. 7, (2006).

[36] L. Bie, A. Rietbrock, S. Hicks, R. Allen, J. Blundy, V. Clouard, J. Collier, J. Davidson, T. Garth, S. Goes, N. Harmon, T. Henstock, J. van Hunen, M. Kendall, F. Krüger, L. Lynch, C. Macpherson, R. Robertson, K. Rychert, S. Tait, J. Wilkinson, M. Wilson, Along-Arc Heterogeneity in Local Seismicity across the Lesser Antilles Subduction Zone from a Dense Ocean-Bottom Seismometer Network. Seismol. Res. Lett. 91, 237–247 (2020).

[37] D. Schlaphorst, J.-M. Kendall, B. Baptie, J. L. Latchman, S. Tait, Gaps, tears and seismic anisotropy around the subducting slabs of the Antilles. Tectonophysics 698, 65–78 (2017).

[38] R. W. Allen, J. S. Collier, A. G. Stewart, T. Henstock, S. Goes, A. Rietbrock; VoiLA Team, The role of arc migration in the development of the Lesser Antilles: A new tectonic model for the Cenozoic evolution of the eastern Caribbean. Geology 47, 891–895 (2019).

[39] R. W. Allen, J. S. Collier, T. J. Henstock, The role of crustal accretion variations in determining slab hydration at an Atlantic subduction zone. J. Geophys. Res. Solid Earth 127, e2022JB024349 (2022).

[40] D. Schlaphorst, J.-M. Kendall, J. S. Collier, J. P. Verdon, J. Blundy, B. Baptie, J. L. Latchman, F. Massin, M.-P. Bouin, Water, oceanic fracture zones and the lubrication of subducting plate boundaries—insights from seismicity. Geophys. J. Int. 204, 1405–1420 (2016).

[41] R. G. Davy, J. S. Collier, T. J. Henstock; VoiLA Consortium, Wide-angle seismic imaging of two modes of crustal accretion in mature Atlantic Ocean crust. J. Geophys. Res. Solid Earth 125, e2019JB019100 (2020).

[42] L. Bie, S. Hicks, A. Rietbrock, S. Goes, J. Collier, C. Rychert, N. Harmon, B. Maunder, Imaging slab-transported fluids and their deep dehydration from seismic velocity tomography in the Lesser Antilles subduction zone. Earth Planet. Sci. Lett. 586, 117535 (2022).

[43] B. Braszus, S. Goes, R. Allen, A. Rietbrock, J. Collier, N. Harmon, T. Henstock, S. Hicks, C. A. Rychert, B. Maunder, J. van Hunen, L. Bie, J. Blundy, G. F. Cooper, R. Davy, J. M. Kendall, C. Macpherson, J. Wilkinson, M. Wilson, Subduction history of the Caribbean from upper-mantle seismic imaging and plate reconstruction. Nat. Commun. 12, 4211 (2021). 34244511 10.1038/s41467-021-24413-0 PMC8270990

[44] N. Harmon, C. A. Rychert, S. Goes, B. Maunder, J. Collier, T. Henstock, L. Lynch, A. Rietbrock; VoiLA Working Group, Widespread hydration of the back arc and the link to variable hydration of the incoming plate in the Lesser Antilles from Rayleigh wave imaging. Geochem. Geophys. Geosyst. 22, e2021GC009707 (2021).

[45] D. Schlaphorst, N. Harmon, J. M. Kendall, C. A. Rychert, J. Collier, A. Rietbrock, S. Goes; VoiLA Team, Variation in upper plate crustal and lithospheric mantle structure in the greater and Lesser Antilles from ambient noise tomography. Geochem. Geophys. Geosyst. 22, e2021GC009800 (2021).

[46] B. Chichester, C. Rychert, N. Harmon, J. Collier, T. Henstock, S. D. B. Goes, J. M. Kendall, F. Krueger, A. Rietbrock, Seismic imaging of the Lesser Antilles subduction zone using S-to-P receiver functions. American Geophysical Union, Fall Meeting 2019, abstract #S53C-0521 (2019).

[47] O. González, V. Clouard, S. Tait, G. F. Panza, S-wave velocities of the lithosphere-asthenosphere system in the Lesser Antilles from the joint inversion of surface wave dispersion and receiver function analysis. Tectonophysics 734–735, 1–15 (2018).

[48] S. Goes, J. Collier, J. Blundy, J. Davidson, N. Harmon, T. Henstock, J. Kendall, C. Macpherson, A. Rietbrock, K. Rychert, J. Prytulak, J. van Hunen, J. J. Wilkinson, M. Wilson, Project VoiLA: Volatile recycling in the Lesser Antilles. Eos 100, (2019).

[49] H. Kopp, W. Weinzierl, A. Becel, P. Charvis, M. Evain, E. R. Flueh, A. Gailler, A. Galve, A. Hirn, A. Kandilarov, D. Klaeschen, M. Laigle, C. Papenberg, L. Planert, E. Roux, Deep structure of the central Lesser Antilles Island Arc: Relevance for the formation of continental crust. Earth Planet. Sci. Lett. 304, 121–134 (2011).

[50] M. Paulatto, M. Laigle, A. Galve, P. Charvis, M. Sapin, G. Bayrakci, M. Evain, H. Kopp, Dehydration of subducting slow-spread oceanic lithosphere in the Lesser Antilles. Nat. Commun. 8, 15980 (2017). 28691714 10.1038/ncomms15980 PMC5508134

[51] I. Jackson, U. H. Faul, Grainsize-sensitive viscoelastic relaxation in olivine: Towards a robust laboratory-based model for seismological application. Phys. Earth Planet. Inter. 183, 151–163 (2010).

[52] C. Havlin, B. K. Holtzman, E. Hopper, Inference of thermodynamic state in the asthenosphere from anelastic properties, with applications to North American upper mantle. Phys. Earth Planet. Inter. 314, 106639 (2021).

[53] U. H. Faul, I. Jackson, Transient creep and strain energy dissipation: An experimental perspective. Annu. Rev. Earth Planet. Sci. 43, 541–569 (2015).

[54] Y. Takei, Effects of partial melting on seismic velocity and attenuation: A new insight from experiments. Annu. Rev. Earth Planet. Sci. 45, 447–470 (2017).

[55] G. A. Abers, P. E. van Keken, B. R. Hacker, The cold and relatively dry nature of mantle forearcs in subduction zones. Nat. Geosci. 10, 333–337 (2017).

[56] J. Corbeau, O. Gonzalez, N. Feuillet, A. Lejeune, F. R. Fontaine, V. Clouard, J. Saurel; OVSM Team, A significant increase in interplate seismicity near major historical earthquakes offshore martinique (FWI). Bull. Seismol. Soc. Am. 111, 3118–3135 (2021).

[57] F. Halpaap, S. Rondenay, A. Perrin, S. Goes, L. Ottemöller, H. Austrheim, R. Shaw, T. Eeken, Earthquakes track subduction fluids from slab source to mantle wedge sink. Sci. Adv. 5, eaav7369 (2019). 30949581 10.1126/sciadv.aav7369 PMC6447373

[58] D. Arcay, Dynamics of interplate domain in subduction zones: Influence of rheological parameters and subducting plate age. Solid Earth 3, 467–488 (2012).

[59] H. Yamauchi, Y. Takei, Polycrystal anelasticity at near-solidus temperatures. J. Geophys. Res. Solid Earth 121, 7790–7820 (2016).

[60] G. Wadge, Comparison of volcanic production rates and subduction rates in the Lesser Antilles and Central America. Geology 12, 555–558 (1984).

[61] M. Bohm, C. Haberland, G. Asch, Imaging fluid-related subduction processes beneath Central Java (Indonesia) using seismic attenuation tomography. Tectonophysics. 590, 175–188 (2013).

[62] T. Chen, R. W. Clayton, Seismic attenuation structure in central Mexico: Image of a focused high-attenuation zone in the mantle wedge. J. Geophys. Res. Solid Earth 114, B07304 (2009).

[63] E. Hopper, H. A. Ford, K. M. Fischer, V. Lekic, M. J. Fouch, The lithosphere–asthenosphere boundary and the tectonic and magmatic history of the northwestern United States. Earth Planet. Sci. Lett. 402, 69–81 (2014).

[64] N. G. Cerpa, I. Wada, C. R. Wilson, Effects of fluid influx, fluid viscosity, and fluid density on fluid migration in the mantle wedge and their implications for hydrous melting. Geosphere 15, 1–23 (2019).

[65] N. Feuillet, I. Manighetti, P. Tapponnier, E. Jacques, Arc parallel extension and localization of volcanic complexes in Guadeloupe, Lesser Antilles. J. Geophys. Res. Solid Earth 107, ETG 3-1–ETG 3-29 (2002).

[66] Institut De Physique Du Globe De Paris (IPGP), Ecole Et Observatoire Des Sciences De La Terre De Strasbourg (EOST), *GEOSCOPE, French Global Network of broad band seismic stations* (IPGP, Université de Paris, 1982);http://geoscope.ipgp.fr/networks/detail/G/.

[67] Institut De Physique Du Globe De Paris (IPGP), *GNSS, seismic broadband and strong motion permanent networks in West Indies* (IPGP, Université de Paris, 2008);http://volobsis.ipgp.fr/networks/detail/WI/.

[68] S. S. Wei, D. A. Wiens, P-wave attenuation structure of the Lau back-arc basin and implications for mantle wedge processes. Earth Planet. Sci. Lett. 502, 187–199 (2018).

[69] K. Aki, P. G. Richards, *Quantitative Seismology* (University Science Books, 2002).

[70] J. G. Anderson, S. E. Hough, A model for the shape of the fourier amplitude spectrum of acceleration at high frequencies. Bull. Seismol. Soc. Am. 74, 1969–1993 (1984).

[71] M. Lindner, A. Rietbrock, L. Bie, S. Goes, J. Collier, C. Rychert, N. Harmon, S. P. Hicks, T. Henstock; VoiLA working group, Bayesian regional moment tensor from ocean bottom seismograms recorded in the Lesser Antilles: Implications for regional stress field. Geophys. J. Int. 233, 1036–1054 (2022).

[72] G. A. Prieto, R. L. Parker, F. L. Vernon III, A Fortran 90 library for multitaper spectrum analysis. Comput. Geosci. 35, 1701–1710 (2009).

[73] R. Madariaga, Dynamics of an expanding circular fault. Bull. Seismol. Soc. Am. 66, 639–666 (1976).

[74] I. Jackson, J. D. F. Gerald, U. H. Faul, B. H. Tan, Grain-size-sensitive seismic wave attenuation in polycrystalline olivine. J. Geophys. Res. Solid Earth 107, ECV 5-1–ECV 5-16 (2002).

[75] D. Eberhart-Phillips, M. Chadwick, S. Bannister, Three-dimensional attenuation structure of central and southern South Island, New Zealand, from local earthquakes. J. Geophys. Res. Solid Earth 113, B05308 (2008).

[76] A. Rietbrock, P wave attenuation structure in the fault area of the 1995 Kobe earthquake. J. Geophys. Res. Solid Earth 106, 4141–4154 (2001).

[77] S. P. Hicks, A. Rietbrock, I. M. Ryder, C.-S. Lee, M. Miller, Anatomy of a megathrust: The 2010 M8.8 Maule, Chile earthquake rupture zone imaged using seismic tomography. Earth Planet. Sci. Lett. 405, 142–155 (2014).

[78] F. Crameri, G. E. Shephard, P. J. Heron, The misuse of colour in science communication. Nat. Commun. 11, 5444 (2020). 33116149 10.1038/s41467-020-19160-7 PMC7595127

[79] L. Krischer, T. Megies, R. Barsch, M. Beyreuther, T. Lecocq, C. Caudron, J. Wassermann, ObsPy: A bridge for seismology into the scientific Python ecosystem. Comput. Sci. Discov. 8, 014003 (2015).

[80] S. Goes, J. Armitage, N. Harmon, H. Smith, R. Huismans, Low seismic velocities below mid-ocean ridges: Attenuation versus melt retention. J. Geophys. Res. Solid Earth 117, B12403 (2012).

