# Supplementary material for: Slab to back-arc to arc: Fluid and melt pathways through the mantle wedge beneath the Lesser Antilles
Source: Sci Adv. 2023 Feb 1;9(5):eadd2143. doi: 10.1126/sciadv.add2143 (PMC9891694; doi:10.1126/sciadv.add2143)
Supplement: Supplementary file 1 — Text S1 Table S1 Figs. S1 to S19 References [file sciadv.add2143_sm.pdf]

Science Advances
AAAS

Supplementary Materials for

Slab to back-arc to arc: Fluid and melt pathways through the mantle wedge beneath the Lesser Antilles

Stephen P. Hicks *et al.*

Corresponding author: Stephen P. Hicks, stephen.hicks@ucl.ac.uk

Sci. Adv. **9**, eadd2143 (2023)
DOI: 10.1126/sciadv.add2143

The PDF file includes:

Text S1
Table S1
Figs. S1 to S19
Legend for data file S1
References

Other Supplementary Material for this manuscript includes the following:

Data file S1

Supplementary Materials

Supplementary Text

Text S1: Seismic attenuation mechanism and grain size effects

In this study, we have mainly interpreted seismic attenuation with the Andrade-pseudoperiod attenuation model of Faul & Jackson (52) (FJ10) (Figure S6). But we also tested the effects of other mechanisms, such as the pre-melting model of Yamauchi & Takei (60) (YT16). We used YT16 to estimate the spatial variation of Q-1 based on our thermal models of the Lesser Antilles subduction zone. We use the parameters that YT16 proposes for matching the oceanic VS from the global Priestley and McKenzie model. Although the predicted attenuation values would vary depending on the tomographic model used to set the parameters, the pattern as a function of temperature will not change. Our model predicts virtually no attenuation (1000/QS ~ 0.1) for most temperatures predicted for the subduction zone (see Figure S17); it reaches a minimum 1000/QS = 7.5 in the core of the mantle wedge when temperatures reach ~90% of a damp mantle solidus. Although pre-melting may be a relevant mechanism, this model alone seems to lack other intrinsic attenuation mechanisms that are important at lower temperatures, and it can not explain most subduction zone Q-1 results. Also with the YT16 model, temperature alone doesn't explain our lowest observed 1000/QS of ~20. We also tested other more temperature-sensitive attenuation models: Qg from Goes et al. (80) and FJ05. Neither of these models are able to explain the lowest Q values with just temperature, unless water significantly enhances temperature sensitivity which has been argued to not be the case (24). The Qg model is less well constrained by experimental data than the FJ10 (52) model (which supersedes FJ05). FJ10 has also been found to be consistent with attenuation measured in mature Pacific oceanic lithosphere at the NoMelt experiment. Therefore, we continue to favour our original interpretation of melt in the mantle wedge.

Figure S18 compares the probability distributions of melt fraction and temperature for the seismic properties of the back-arc mantle wedge of Dominica using the Andrade-pseudoperiod model (52) and the pre-melting model of YT16 in the Very Broadband Rheology (VBR) calculator (53). The pre-melting model results in two probable temperature-melt fields: either high temperature (~1500°C) with a very low melt fraction or a lower temperature of ~1300°C with a melt fraction similar to that predicted by the Andrade-pseudoperiod model (~0.02). Given the predicted maximum mantle wedge temperature of ~1350°C in our thermal models for the Lesser Antilles subduction zone, we prefer the higher melt fraction model.

Although the joint V, Q distribution exhibits a very weak preference for larger grain size and although the grain size effect produces only small differences in the predicted Q-1 structure, we opted for a 1 mm grain size in our preferred thermal model in Figure S6. To investigate further, we imposed two different log-normal prior models of 1mm & 1cm in VBR, and we can see some differences in temperature - melt fraction space (Figure S19). The larger a priori grain size requires a higher temperature of ~1400°C and smaller melt fraction of ~0.01-0.02.

Supplementary Tables

**Cluster #**	**Spatial domain**	**V_P (km/s)**	**V_{SV} (km/s)**	**V_P/V_S**	**1000/Q_S**	**Q_κ^{-1}/Q_μ^{-1}**
1	Backarc asthenosphere mantle (less attenuating)	7.86	4.40	1.78	8	0.8
2	Cold boundary layer and slab	8.09	4.50	1.78	4	13.9
3	Forearc mantle corner and slab	7.91	4.44	1.77	4	1.3
4	Backarc asthenosphere mantle (more attenuating)	7.94	4.43	1.79	13	0.6

Table S1: Centroid seismic properties based on *k*-means clustering using 4 clusters.

Supplementary Figures

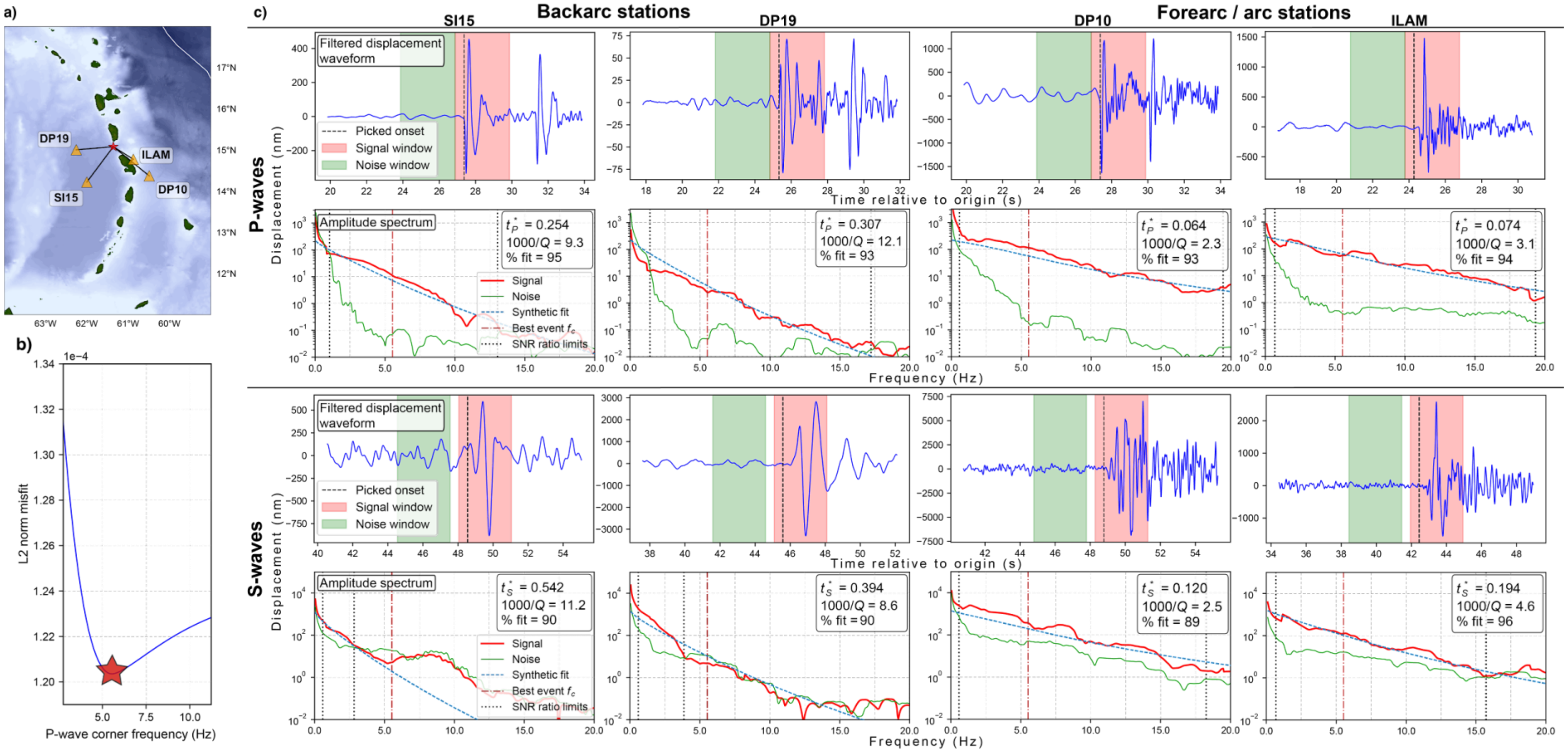

Figure S1. Example of t^* inversions for an M_L 4.1 event on 2016-09-08T21:39 at 182 km depth. a) Map shows the event and station locations with spectra plotted in c). b) t^* inversion misfit as a function of P-wave corner frequency. The red star gives the best-fitting corner frequency used for computing t^*. c) Each sub-panel comprises the displacement waveforms (top) and spectra (bottom). The top row shows *P*-waves; the bottom shows *S*-waves. Stations SI15 are DP19 are OBSs in the backarc; DP10 is an OBS in the forearc; ILAM is an onshore station on the volcanic arc.

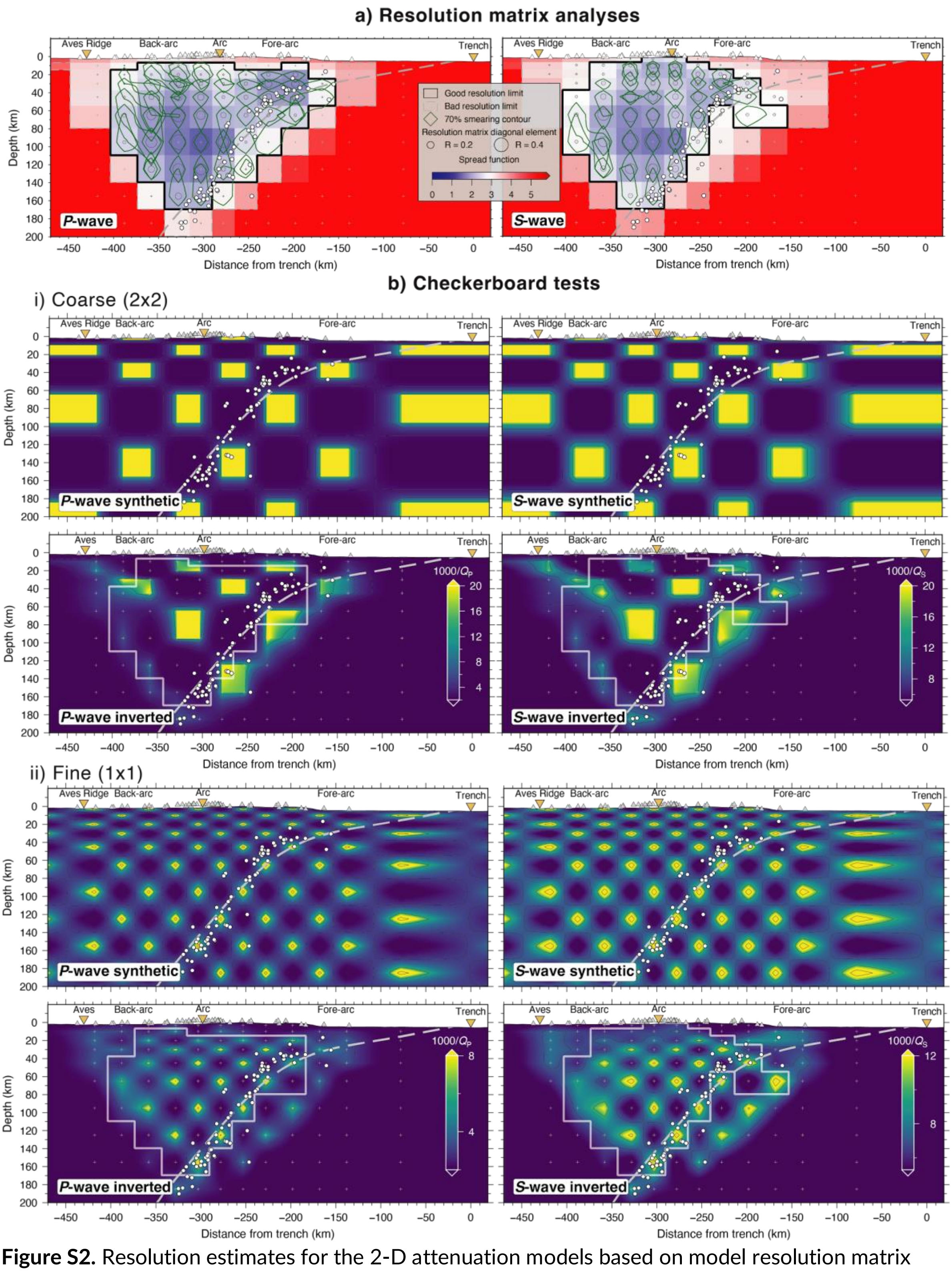

Figure S2. Resolution estimates for the 2-D attenuation models based on model resolution matrix analyses (a) and checkerboard tests (b) *P*-wave results are shown in the left-hand columns; *S*-waves on the right. The cross-sections correspond to A-A' shown in Figure 1. In (a), low spread values and symmetric smearing contours indicate regions of the best resolution. We use two checkerboards: a coarse and a fine one. The thick white line shows the good resolution limit from (a). Small white crosses are model inversion nodes. White circles are event hypocentres; white triangles are the locations of seismic stations projected onto the cross-section.

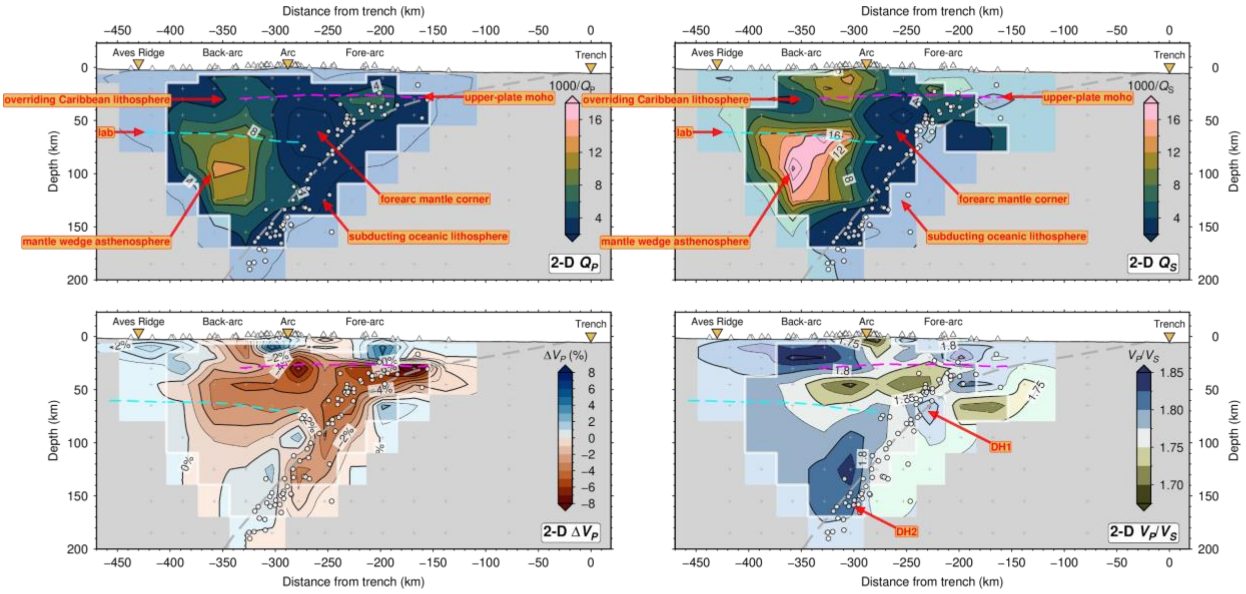

Figure S3. Same as Figure 2, but showing the relative changes in P-wave velocity relative to a 1-D reference model (*37*) in the bottom-left panel.

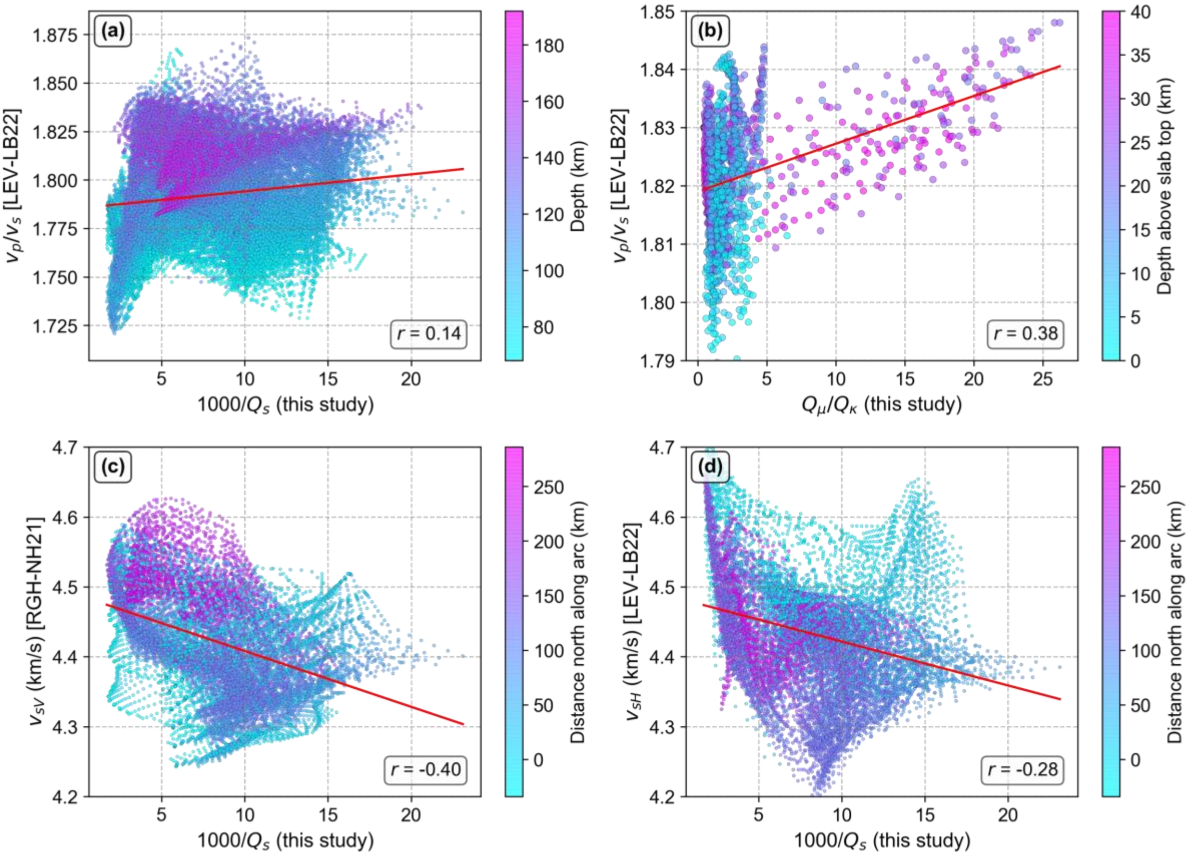

Figure S4. Cross-plots comparing seismic attenuation and velocities. The top row shows V_P/V_S from LEV-LB22 (*43*) plotted against (a) 1000/Q_S in the mantle wedge and (b) Q_μ/Q_κ at 140-160 km depth in the wedge. The bottom row shows wedge *S*-wave velocities from (c) teleseismic rayleigh wave imaging from RLH-NH21 (*45*) and (d) from V_P and V_P/V_S in LEV-LB22, plotted against 1000/Q_S. The *r*-value labelled gives the Pearson correlation coefficient. Scatter points are sampled from the 3-D attenuation and velocity models using a 4 km spacing. Red lines show linear fits to the data.

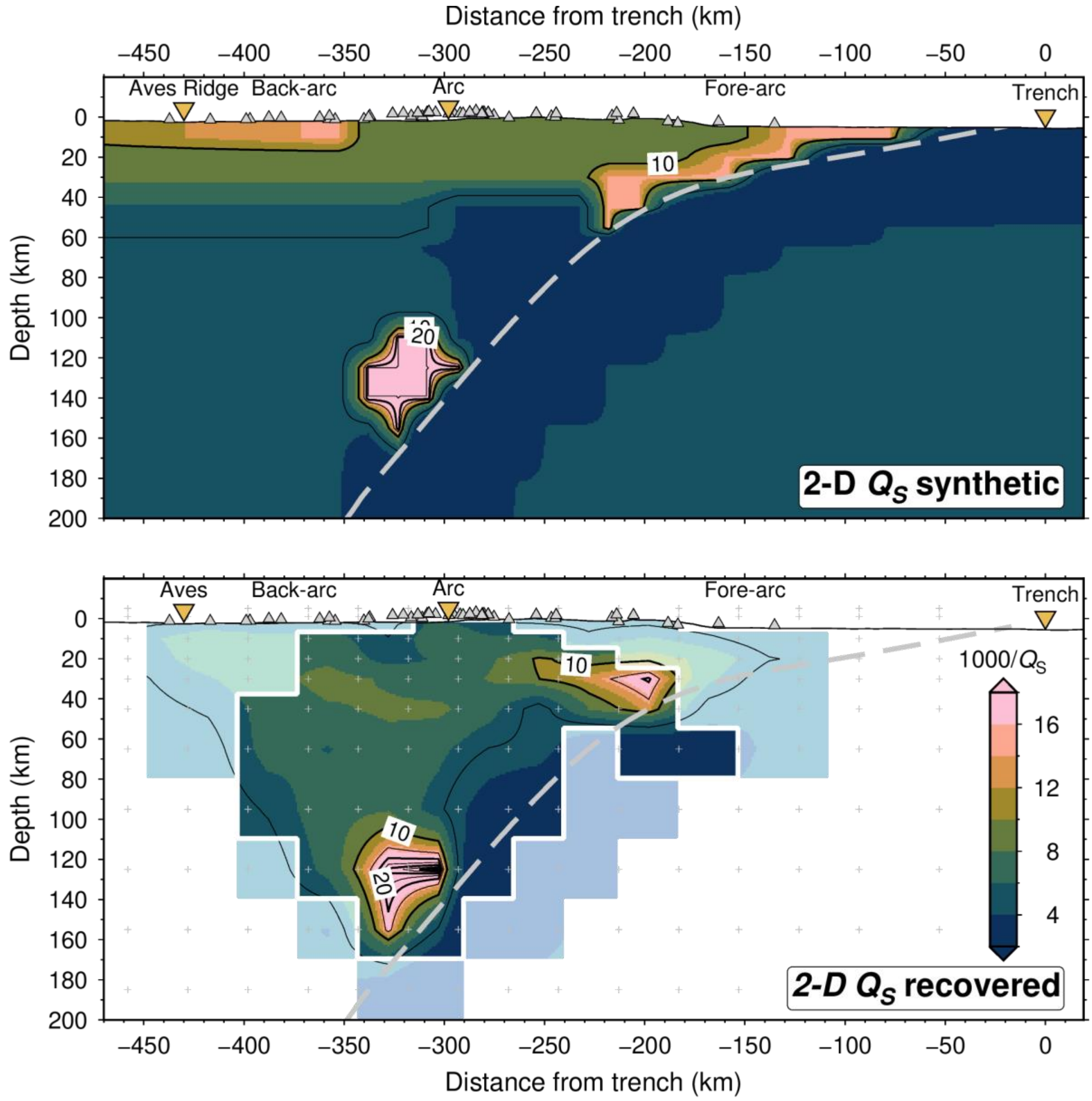

Figure S5: Similar plot to Figure 5a, showing a synthetic restoring resolution test for the 2-D attenuation model. This test explores whether a high 1/Q anomaly in the location of the observed high Vp/Vs anomaly is resolvable.

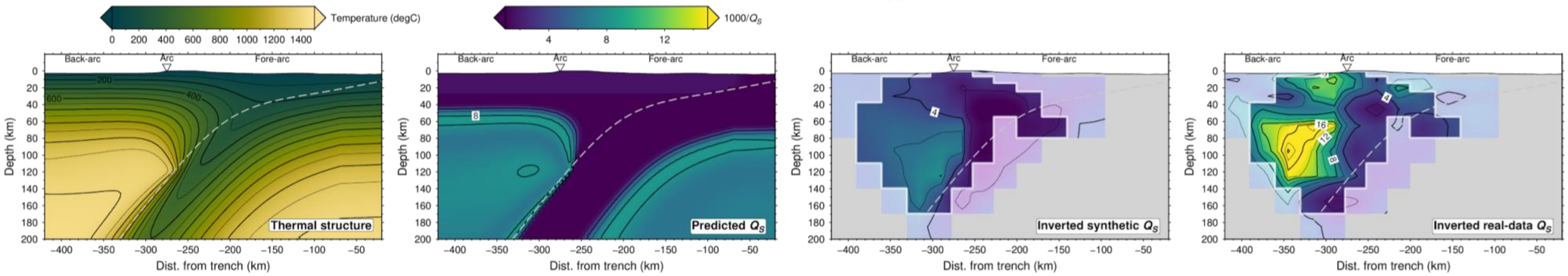

Figure S6. Left: predicted 2-D thermal structure beneath Dominica along the LAA using a decoupling depth of 120 km, plate convergence velocity of 2 cm/yr, and a grain size of 1 mm. Second panel: the predicted Q_s structure based on the thermal model using the FJ10 attenuation model (*52*). Third panel: recovered synthetic Q_s structure after inverting synthetic t^* data. Fourth panel: comparison to the 2-D Q_s model using the real data as presented in Figure 2.

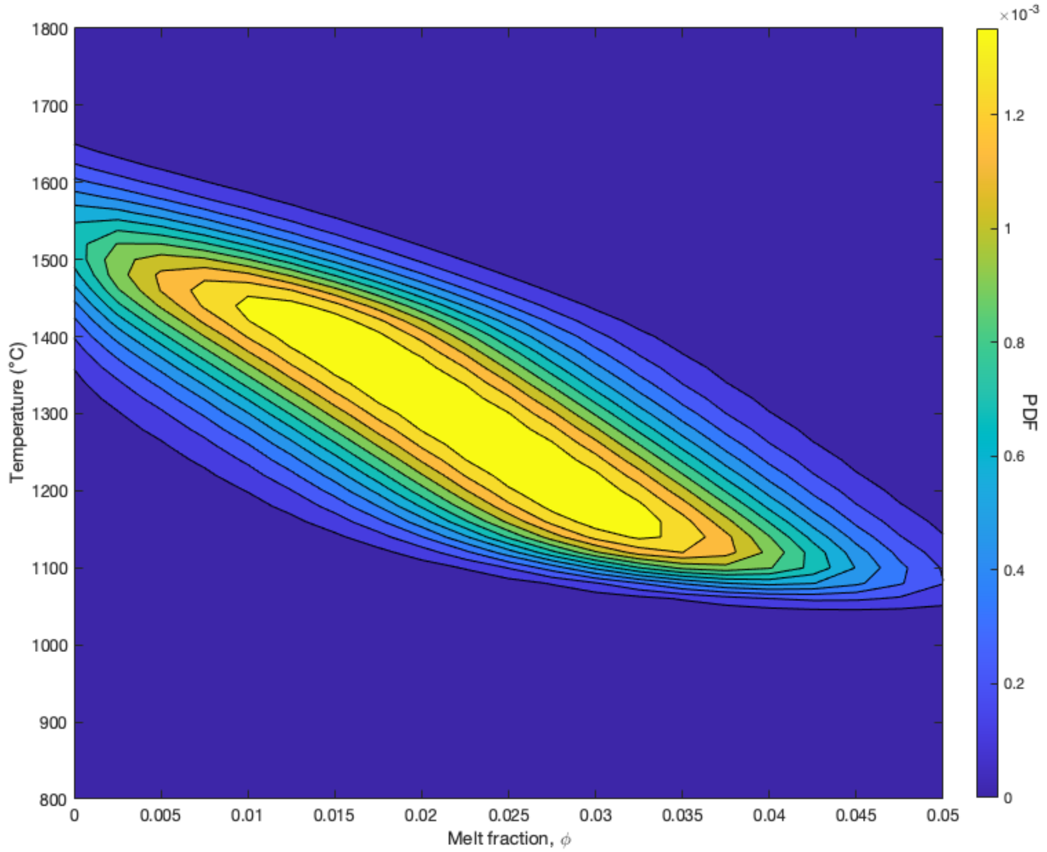

Figure S7. Probability distributions of melt fraction and temperature for the back-arc mantle wedge of Dominica. We use an ensemble weight of the joint probability distribution for two anelastic methods: the Andrade-pseudoperiod and modified Burgers models (*52*). We use the depth range of 65 km to 105 km compute averaged representative seismic properties (1000/Q_S = 16; V_S = 4.3 km/s).

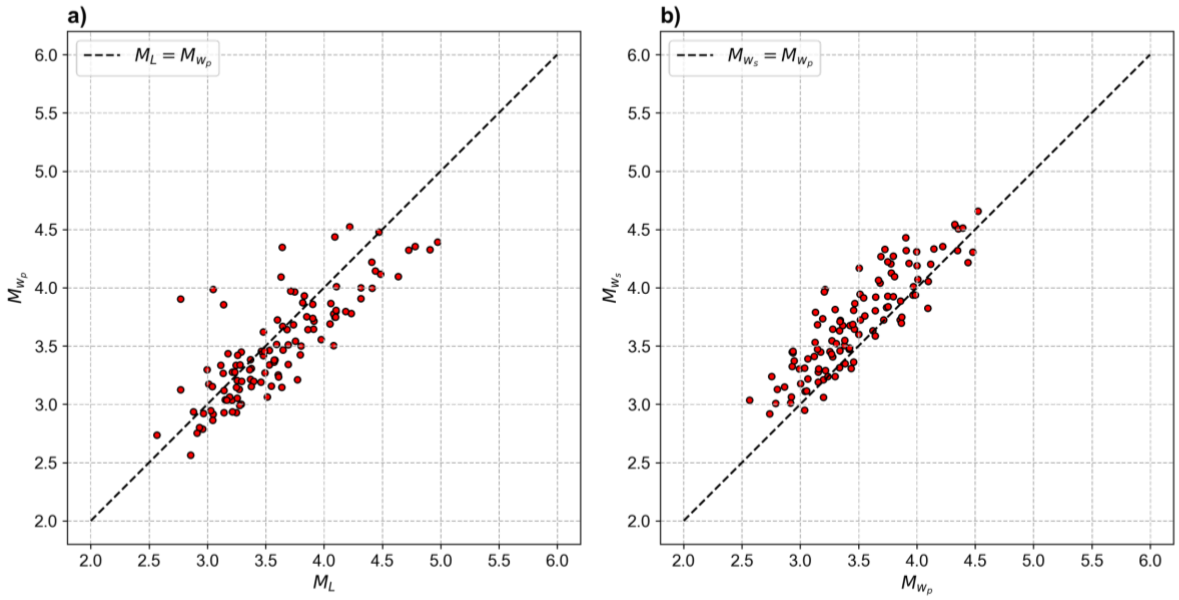

Figure S8: Event magnitudes. a) Comparison between local magnitude from our input catalogue (*37*) with our computed moment magnitudes from P-wave spectra. b) Comparison between moment magnitude from P- and S-wave spectra, respectively. In both cases, the dashed black line shows a 1:1 relationship.

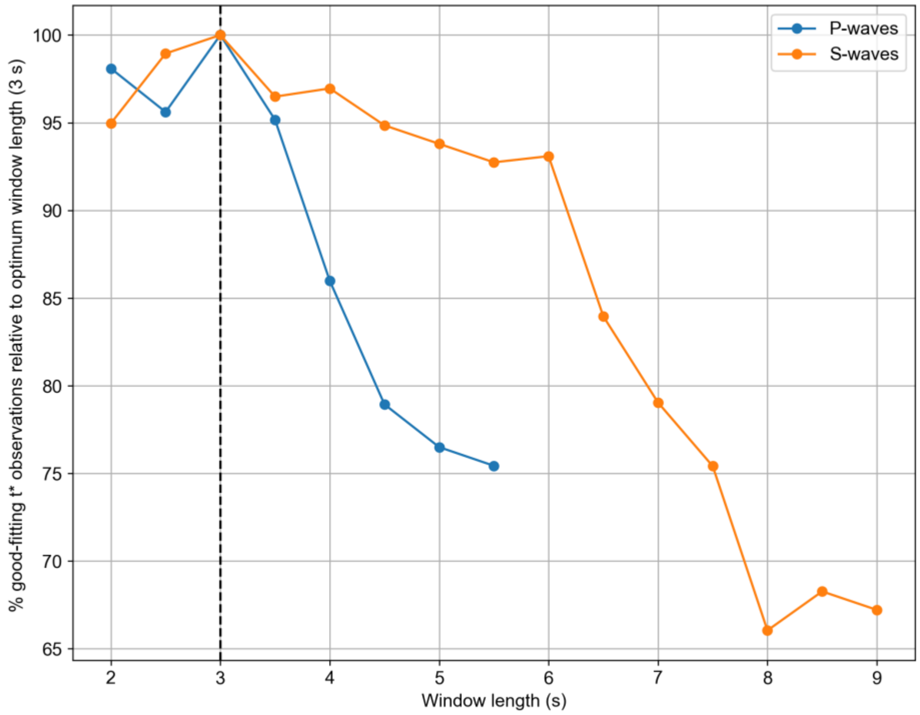

Figure S9: The effect of window length on the number of good-fitting t^* observations retrieved. The dashed black line indicates the optimum window length used of 3.0 s for both P- and S-waves.

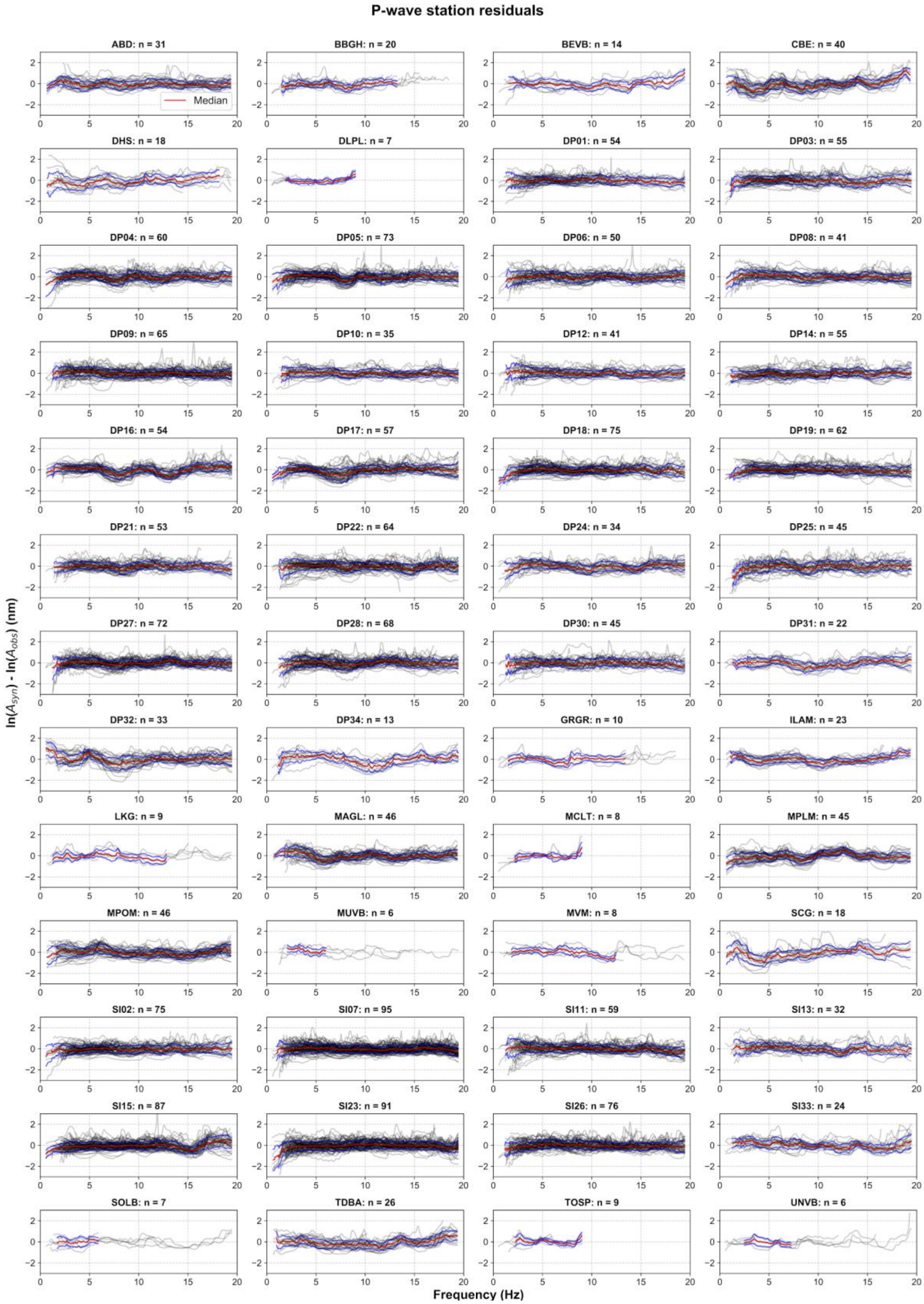

Figure S10: *P*-wave site effects of all stations that have at least 6 observations. For each panel, each grey line indicates the residual spectrum for each t^* observation. The red line shows the overall site effect, which is the median of all residual spectra. The blue line shows the standard deviation from the median. The number given after the station name (*n*) in the title for each subplot shows the number of traces used for that site. Station names with the prefix DP and SI are OBSs; others are land stations.

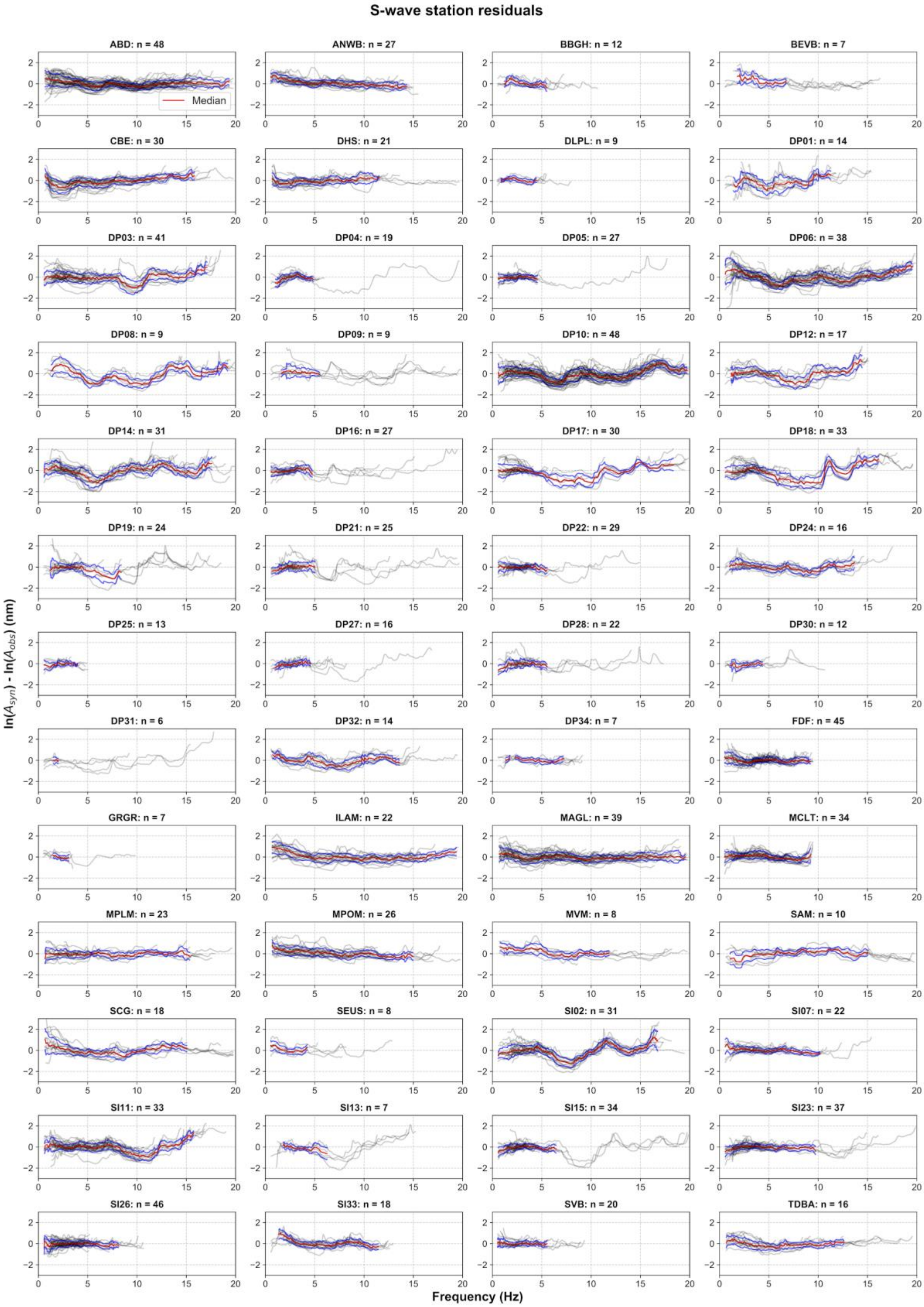

Figure S11: *S*-wave site effects of all stations. This figure is plotted in the same way as Figure S10.

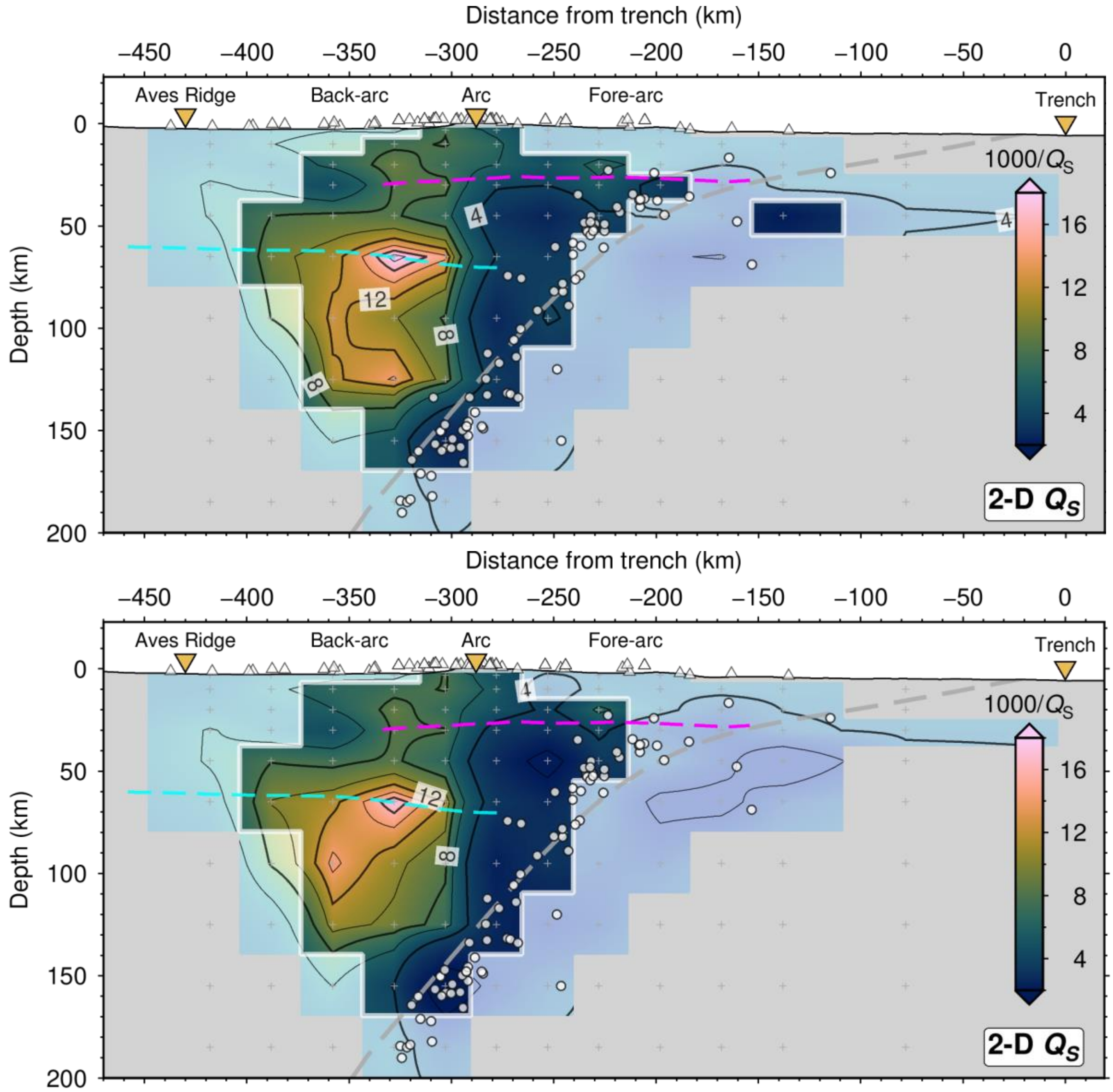

Figure S12. Testing the effects of key assumptions on the 2-D tomographic inversion for Q_s. The top panel shows the inversion result if we do not remove the average station spectra (Figure S11) when computing t* values. The bottom panel shows the inversion result if we assume S-wave corner frequency, $f_{cs} = f_{cp}/1.5$, compared to our main inversion in which $f_{cs} = f_{cp}$.

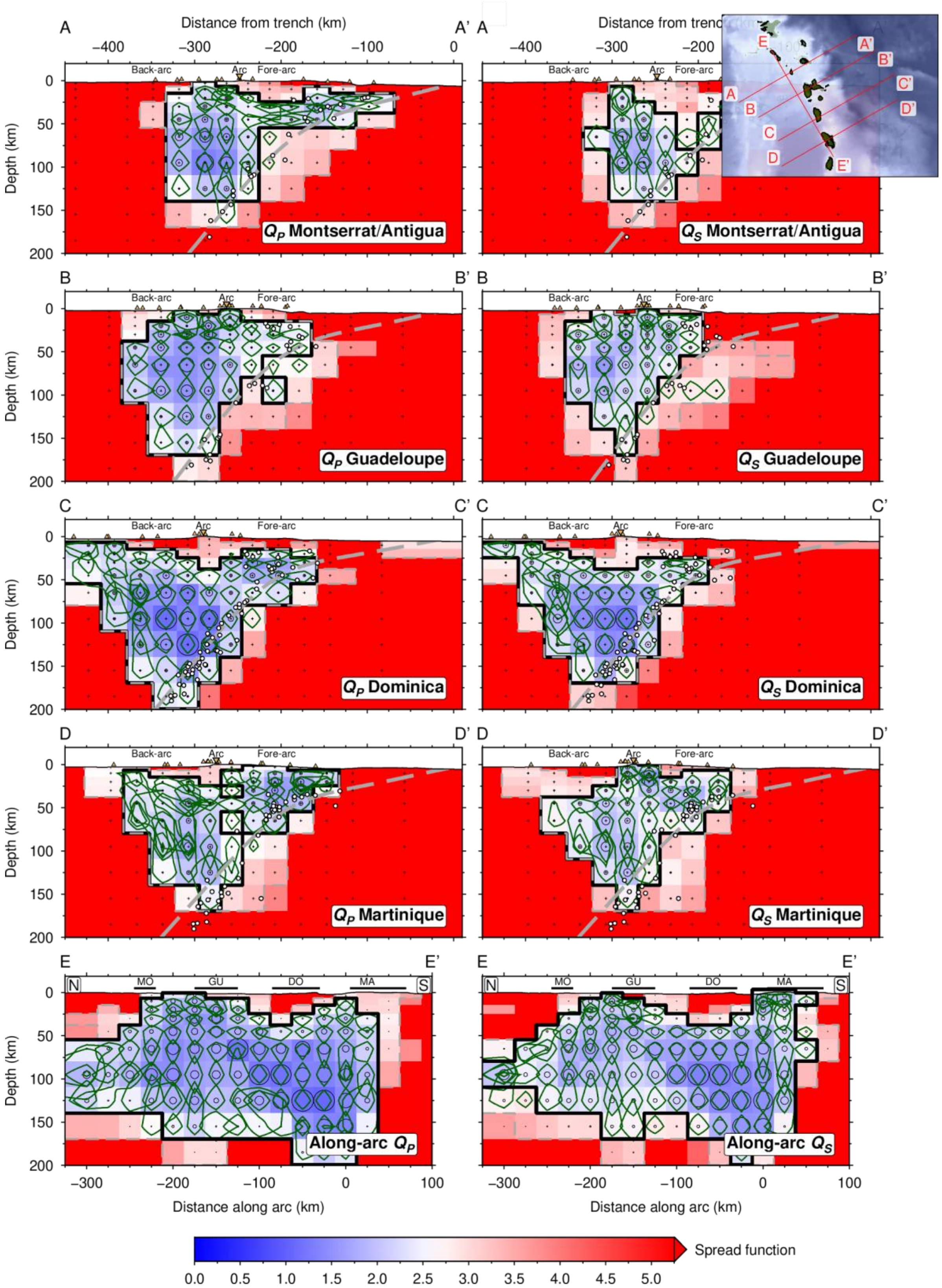

Figure S13. Resolution estimates for the 2-D attenuation models based on model resolution matrix analyses, plotted in the same way as Figure S2a. Locations of each cross-section are shown in the inset map on the top-right.

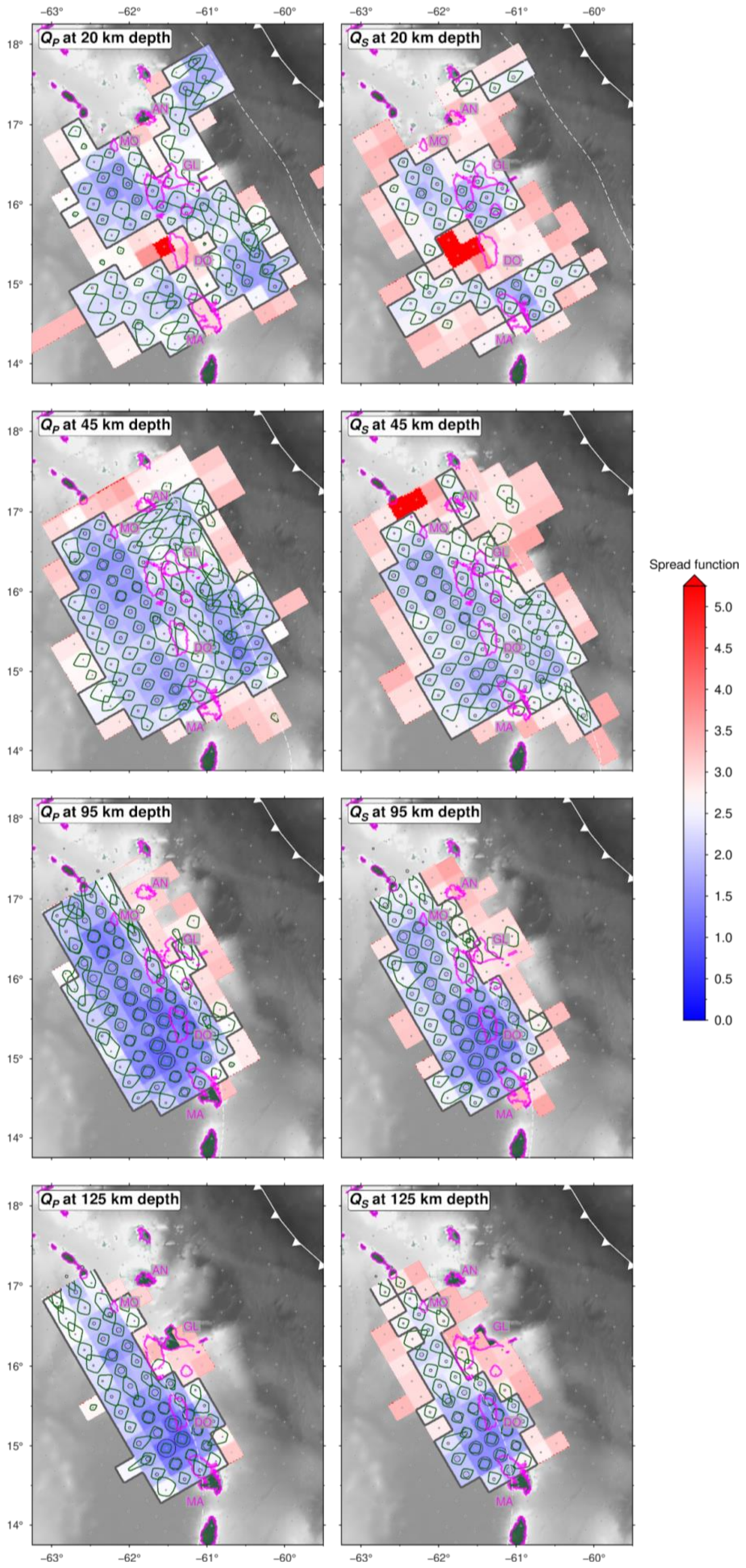

Figure S14. Same as Figure S2a, but plotted as horizontal depth sections.

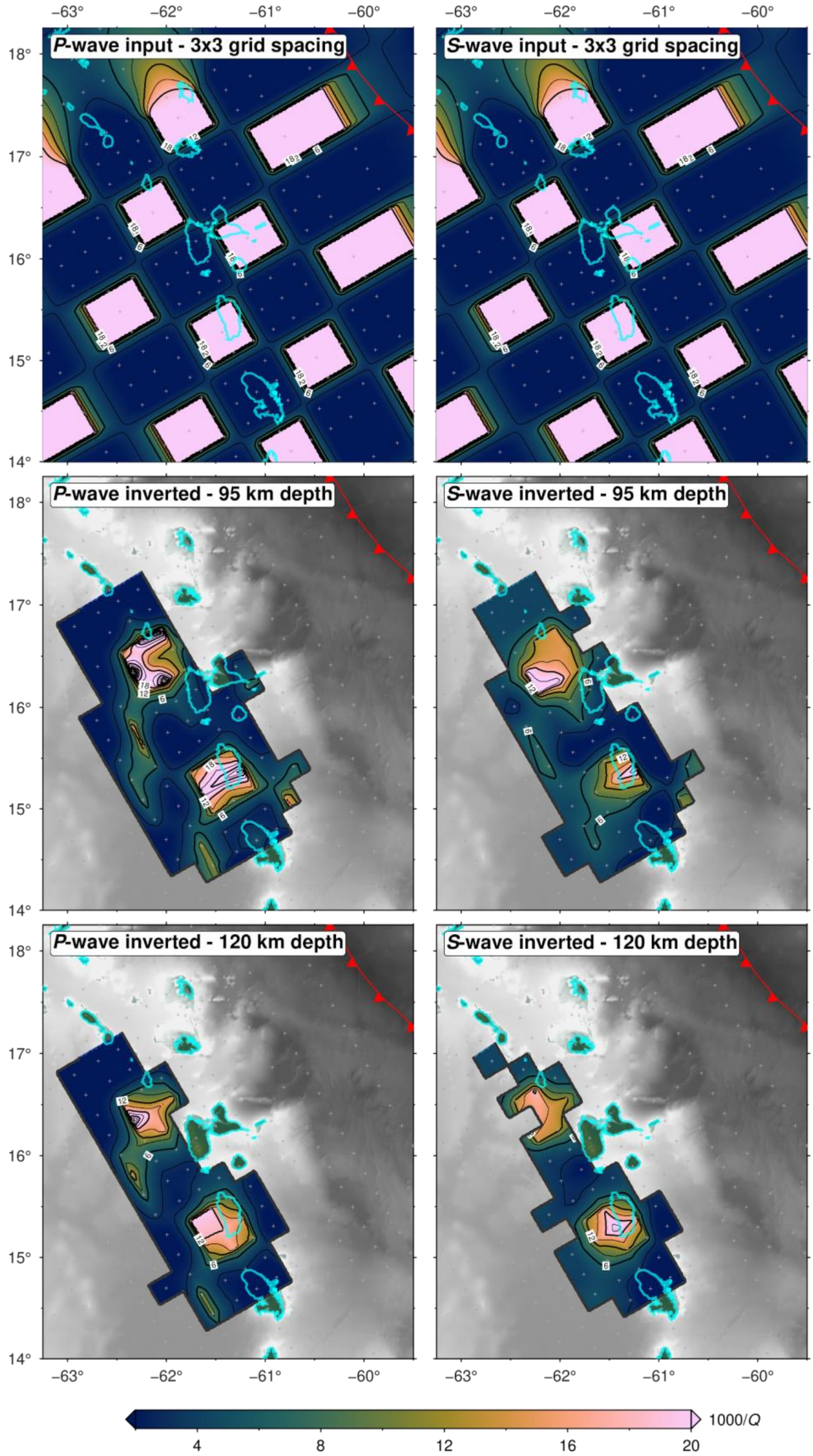

Figure S15. Checkerboard test for the 3-D inversion using coarse anomalies (minimum 75 km dimension) shown as horizontal depth sections. The top row shows the input pattern and the lower two rows show the recovery, which is only shown where resolution is good, based on formal resolution matrix analysis (Figures S13-S14).

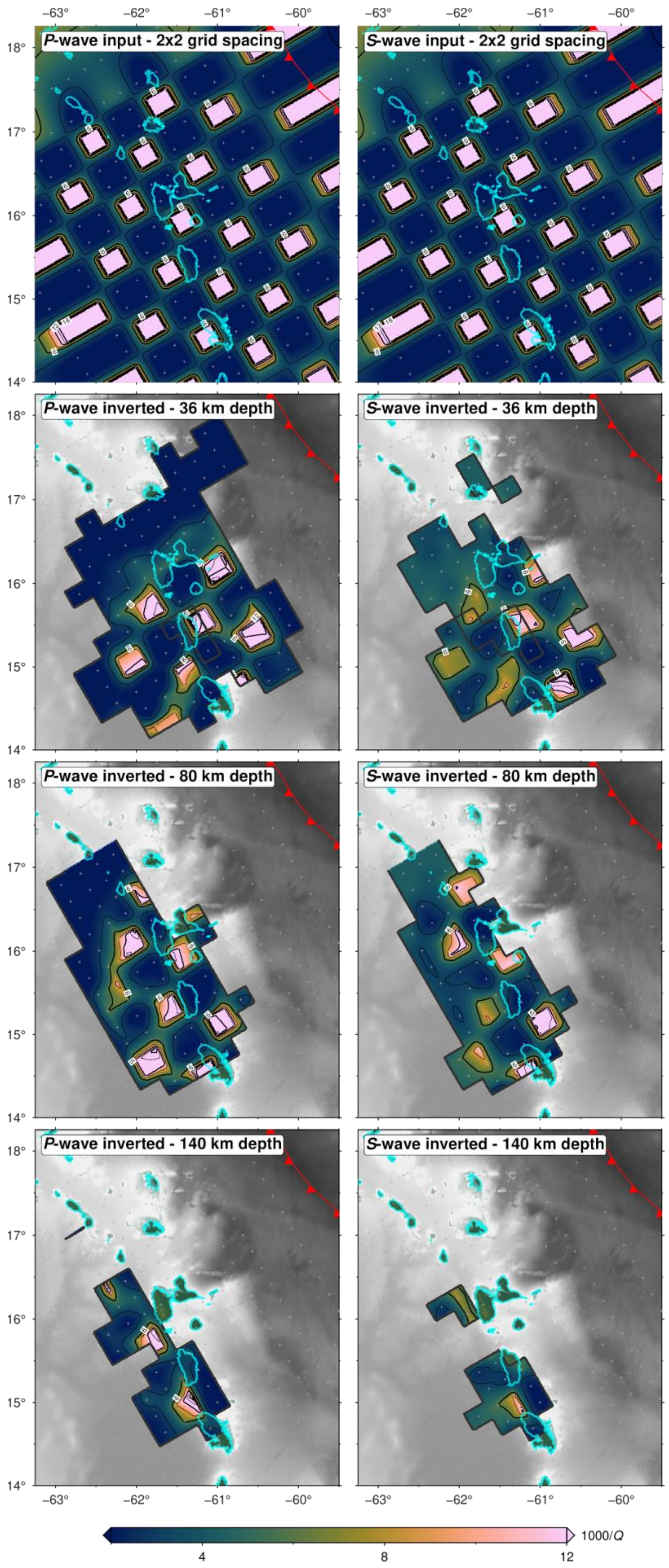

Figure S16. Checkerboard test for the 3-D inversion using fine anomalies (minimum 50 km dimension) shown as horizontal depth sections, plotted in the same way as Figure S15.

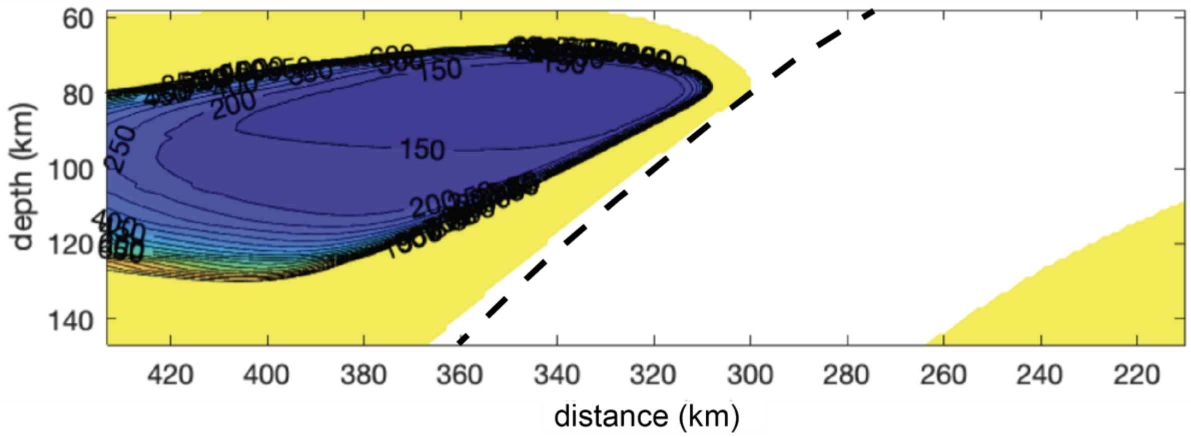

Figure S17. Predicted Q structure (similar to second panel in Figure S6) using the pre-melting attenuation model of Yamauchi & Takei (60). See Text S1 for details on the modelling parameters. The black dashed line indicates the top of the subducting plate.

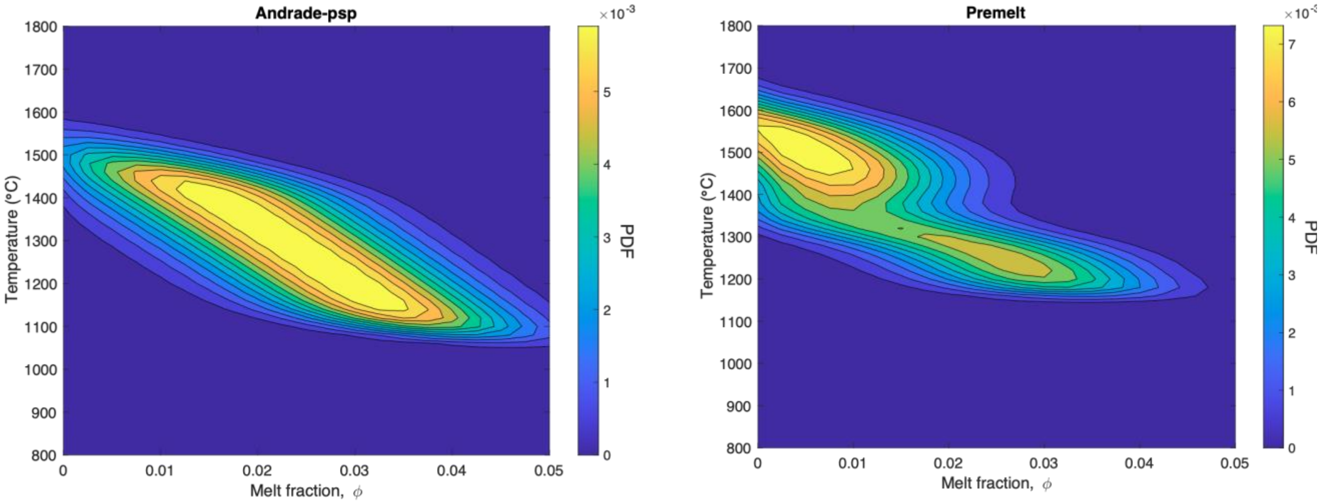

Figure S18. Same as Figure S7, but instead showing the individual probability density functions of temperature and melt fraction individually for the Andrade pseudoperiod attenuation model (52) (left) and the premelting model (60) (right).

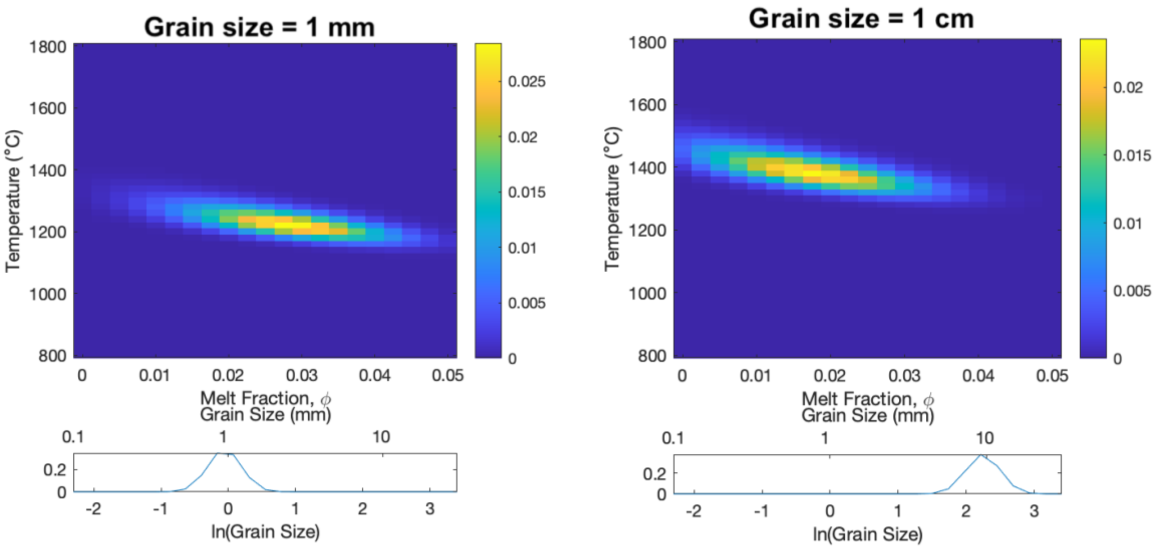

Figure S19. Joint probability density function of temperature and melt fraction (top panels; similar to Figure S7) using Andrade pseudoperiod scaling and assuming a log-normal distribution for the prior model of grain size (lower panels), with a grain size of 1 mm on the left and 1 cm on the right.

Supplementary Data Files

Data File S1: Other VoiLA (Volatiles in the Lesser Antilles) consortium members.

REFERENCES AND NOTES

1. R. J. Stern, Subduction Zones. *Rev. Geophys.* **40**, 3-1–3–38 (2002).

2. E. M. Syracuse, P. E. van Keken, G. A. Abers, The global range of subduction zone thermal models. *Phys. Earth Planet. Inter.* **183**, 73–90 (2010).

3. I. Wada, K. Wang, Common depth of slab-mantle decoupling: Reconciling diversity and uniformity of subduction zones. *Geochem. Geophys. Geosyst.* **10**, Q10009 (2009).

4. C. B. Till, A. J. R. Kent, G. A. Abers, H. A. Janiszewski, J. B. Gaherty, B. W. Pitcher, The causes of spatiotemporal variations in erupted fluxes and compositions along a volcanic arc. *Nat. Commun.* **10**, 1350 (2019).

5. A. Perrin, S. Goes, J. Prytulak, D. R. Davies, C. Wilson, S. Kramer, Reconciling mantle wedge thermal structure with arc lava thermobarometric determinations in oceanic subduction zones. *Geochem. Geophys. Geosyst.* **17**, 4105–4127 (2016).

6. H. Andikagumi, C. G. Macpherson, K. J. W. McCaffrey, Upper plate stress controls the distribution of Mariana arc volcanoes. *J. Geophys. Res. Solid Earth* **125**, e2019JB017391 (2020).

7. G. A. Abers, P. E. van Keken, C. R. Wilson, Deep decoupling in subduction zones: Observations and temperature limits. *Geosphere* **16**, 1408–1424 (2020).

8. E. M. Syracuse, G. A. Abers, Global compilation of variations in slab depth beneath arc volcanoes and implications. *Geochem. Geophys. Geosyst.* **7**, Q05017 (2006).

9. B. C. Kerswell, M. J. Kohn, T. V. Gerya, Backarc lithospheric thickness and serpentine stability control slab-mantle coupling depths in subduction zones. *Geochem. Geophys. Geosyst.* **22**, e2020GC009304 (2021).

10. N. Harmon, D. K. Blackman, Effects of plate boundary geometry and kinematics on mantle melting beneath the back-arc spreading centers along the Lau Basin. *Earth Planet. Sci. Lett.* **298**, 334–346 (2010).

11. H. Iwamori, Transportation of H_2O and melting in subduction zones. *Earth Planet. Sci. Lett.* **160**, 65–80 (1998).

12. J. H. Davies, D. J. Stevenson, Physical model of source region of subduction zone volcanics. *J. Geophys. Res. Solid Earth* **97**, 2037–2070 (1992).

13. L. B. Cooper, D. M. Ruscitto, T. Plank, P. J. Wallace, E. M. Syracuse, C. E. Manning, Global variations in H_2O/Ce: 1. Slab surface temperatures beneath volcanic arcs. *Geochem. Geophys. Geosyst.* **13**, Q03024 (2012).

14. N. G. Cerpa, I. Wada, C. R. Wilson, Fluid migration in the mantle wedge: Influence of mineral grain size and mantle compaction. *J. Geophys. Res. Solid Earth* **122**, 6247–6268 (2017).

15. C. R. Wilson, M. Spiegelman, P. E. van Keken, B. R. Hacker, Fluid flow in subduction zones: The role of solid rheology and compaction pressure. *Earth Planet. Sci. Lett.* **401**, 261–274 (2014).

16. G. Ha, L. G. J. Montési, W. Zhu, Melt focusing along permeability barriers at subduction zones and the location of volcanic arcs. *Geochem. Geophys. Geosyst.* **21**, e2020GC009253 (2020).

17. G. F. Cooper, C. G. Macpherson, J. D. Blundy, B. Maunder, R. W. Allen, S. Goes, J. S. Collier, L. Bie, N. Harmon, S. P. Hicks, A. A. Iveson, J. Prytulak, A. Rietbrock, C. A. Rychert, J. P. Davidson; VoiLA team, Variable water input controls evolution of the Lesser Antilles volcanic arc. *Nature* **582**, 525–529 (2020).

18. C. Kincaid, I. S. Sacks, Thermal and dynamical evolution of the upper mantle in subduction zones. *J. Geophys. Res. Solid Earth* **102**, 12295–12315 (1997).

19. P. C. England, R. F. Katz, Melting above the anhydrous solidus controls the location of volcanic arcs. *Nature* **467**, 700–703 (2010).

20. A.-M. Cagnioncle, E. M. Parmentier, L. T. Elkins-Tanton, Effect of solid flow above a subducting slab on water distribution and melting at convergent plate boundaries. *J. Geophys. Res. Solid Earth* **112**, B09402 (2007).

21. S. Rondenay, L. G. J. Montési, G. A. Abers, New geophysical insight into the origin of the Denali volcanic gap. *Geophys. J. Int.* **182**, 613–630 (2010).

22. B. R. Jicha, S. M. Kay, Quantifying arc migration and the role of forearc subduction erosion in the central Aleutians. *J. Volcanol. Geotherm. Res.* **360**, 84–99 (2018).

23. G. A. Abers, K. M. Fischer, G. Hirth, D. A. Wiens, T. Plank, B. K. Holtzman, C. McCarthy, E. Gazel, Reconciling mantle attenuation-temperature relationships from seismology, petrology, and laboratory measurements. *Geochem. Geophys. Geosyst.* **15**, 3521–3542 (2014).

24. D. Eberhart-Phillips, S. Bannister, M. Reyners, Attenuation in the mantle wedge beneath super-volcanoes of the Taupo Volcanic Zone, New Zealand. *Geophys. J. Int.* **220**, 703–723 (2020).

25. S. H. Pozgay, D. A. Wiens, J. A. Conder, H. Shiobara, H. Sugioka, Seismic attenuation tomography of the Mariana subduction system: Implications for thermal structure, volatile distribution, and slow spreading dynamics. *Geochem. Geophys. Geosyst.* **10**, Q04X05 (2009).

26. S. S. Wei, D. A. Wiens, High bulk and shear attenuation due to partial melt in the Tonga-Lau back-arc mantle. *J. Geophys. Res. Solid Earth* **125**, e2019JB017527 (2020).

27. C. A. Rychert, K. M. Fischer, G. A. Abers, T. Plank, E. Syracuse, J. M. Protti, V. Gonzalez, W. Strauch, Strong along-arc variations in attenuation in the mantle wedge beneath Costa Rica and Nicaragua. *Geochem. Geophys. Geosyst.* **9**, Q10S10 (2008).

28. J. C. Stachnik, G. A. Abers, D. H. Christensen, Seismic attenuation and mantle wedge temperatures in the Alaska subduction zone. *J. Geophys. Res. Solid Earth* **109**, B10304 (2004).

29. B. Budiansky, E. E. Sumner Jr., R. J. O’Connell, Bulk thermoelastic attenuation of composite materials. *J. Geophys. Res. Solid Earth* **88**, 10343–10348 (1983).

30. X. Liu, D. Zhao, S. Li, Seismic attenuation tomography of the Northeast Japan arc: Insight into the 2011 Tohoku earthquake (Mw 9.0) and subduction dynamics. *J. Geophys. Res. Solid Earth* **119**, 1094–1118 (2014).

31. J. Nakajima, S. Hada, E. Hayami, N. Uchida, A. Hasegawa, S. Yoshioka, T. Matsuzawa, N. Umino, Seismic attenuation beneath northeastern Japan: Constraints on mantle dynamics and arc magmatism. *J. Geophys. Res. Solid Earth* **118**, 5838–5855 (2013).

32. B. Schurr, G. Asch, A. Rietbrock, R. Trumbull, C. Haberland, Complex patterns of fluid and melt transport in the central Andean subduction zone revealed by attenuation tomography. *Earth Planet. Sci. Lett.* **215**, 105–119 (2003).

33. E. M. Syracuse, G. A. Abers, K. Fischer, L. MacKenzie, C. Rychert, M. Protti, V. González, W. Strauch, Seismic tomography and earthquake locations in the Nicaraguan and Costa Rican upper mantle. *Geochem. Geophys. Geosyst.* **9**, Q07S08 (2008).

34. M. Reyners, D. Eberhart-Phillips, G. Stuart, Y. Nishimura, Imaging subduction from the trench to 300 km depth beneath the central North Island, New Zealand, with *Vp* and *V*p/*V*s. *Geophys. J. Int.* **165**, 565–583 (2006).

35. J. A. Conder, D. A. Wiens, Seismic structure beneath the Tonga arc and Lau back-arc basin determined from joint *Vp, Vp*/*Vs* tomography. *Geochem. Geophys. Geosyst.* **7** (2006).

36. L. Bie, A. Rietbrock, S. Hicks, R. Allen, J. Blundy, V. Clouard, J. Collier, J. Davidson, T. Garth, S. Goes, N. Harmon, T. Henstock, J. van Hunen, M. Kendall, F. Krüger, L. Lynch, C. Macpherson, R. Robertson, K. Rychert, S. Tait, J. Wilkinson, M. Wilson, Along-Arc Heterogeneity in Local Seismicity across the Lesser Antilles Subduction Zone from a Dense Ocean-Bottom Seismometer Network. *Seismol. Res. Lett.* **91**, 237–247 (2020).

37. D. Schlaphorst, J.-M. Kendall, B. Baptie, J. L. Latchman, S. Tait, Gaps, tears and seismic anisotropy around the subducting slabs of the Antilles. *Tectonophysics* **698**, 65–78 (2017).

38. R. W. Allen, J. S. Collier, A. G. Stewart, T. Henstock, S. Goes, A. Rietbrock; VoiLA Team, The role of arc migration in the development of the Lesser Antilles: A new tectonic model for the Cenozoic evolution of the eastern Caribbean. *Geology* **47**, 891–895 (2019).

39. R. W. Allen, J. S. Collier, T. J. Henstock, The role of crustal accretion variations in determining slab hydration at an Atlantic subduction zone. *J. Geophys. Res. Solid Earth* **127**, e2022JB024349 (2022).

40. D. Schlaphorst, J.-M. Kendall, J. S. Collier, J. P. Verdon, J. Blundy, B. Baptie, J. L. Latchman, F. Massin, M.-P. Bouin, Water, oceanic fracture zones and the lubrication of subducting plate boundaries—insights from seismicity. *Geophys. J. Int.* **204**, 1405–1420 (2016).

41. R. G. Davy, J. S. Collier, T. J. Henstock; VoiLA Consortium, Wide-angle seismic imaging of two modes of crustal accretion in mature Atlantic Ocean crust. *J. Geophys. Res. Solid Earth* **125**, e2019JB019100 (2020).

42. L. Bie, S. Hicks, A. Rietbrock, S. Goes, J. Collier, C. Rychert, N. Harmon, B. Maunder, Imaging slab-transported fluids and their deep dehydration from seismic velocity tomography in the Lesser Antilles subduction zone. *Earth Planet. Sci. Lett.* **586**, 117535 (2022).

43. B. Braszus, S. Goes, R. Allen, A. Rietbrock, J. Collier, N. Harmon, T. Henstock, S. Hicks, C. A. Rychert, B. Maunder, J. van Hunen, L. Bie, J. Blundy, G. F. Cooper, R. Davy, J. M. Kendall, C. Macpherson, J. Wilkinson, M. Wilson, Subduction history of the Caribbean from upper-mantle seismic imaging and plate reconstruction. *Nat. Commun.* **12**, 4211 (2021).

44. N. Harmon, C. A. Rychert, S. Goes, B. Maunder, J. Collier, T. Henstock, L. Lynch, A. Rietbrock; VoiLA Working Group, Widespread hydration of the back arc and the link to variable hydration of the incoming plate in the Lesser Antilles from Rayleigh wave imaging. *Geochem. Geophys. Geosyst.* **22**, e2021GC009707 (2021).

45. D. Schlaphorst, N. Harmon, J. M. Kendall, C. A. Rychert, J. Collier, A. Rietbrock, S. Goes; VoiLA Team, Variation in upper plate crustal and lithospheric mantle structure in the greater and Lesser Antilles from ambient noise tomography. *Geochem. Geophys. Geosyst.* **22**, e2021GC009800 (2021).

46. B. Chichester, C. Rychert, N. Harmon, J. Collier, T. Henstock, S. D. B. Goes, J. M. Kendall, F. Krueger, A. Rietbrock, Seismic imaging of the Lesser Antilles subduction zone using S-to-P receiver functions. American Geophysical Union, Fall Meeting 2019, abstract #S53C-0521 (2019).

47. O. González, V. Clouard, S. Tait, G. F. Panza, S-wave velocities of the lithosphere-asthenosphere system in the Lesser Antilles from the joint inversion of surface wave dispersion and receiver function analysis. *Tectonophysics* **734–735**, 1–15 (2018).

48. S. Goes, J. Collier, J. Blundy, J. Davidson, N. Harmon, T. Henstock, J. Kendall, C. Macpherson, A. Rietbrock, K. Rychert, J. Prytulak, J. van Hunen, J. J. Wilkinson, M. Wilson, Project VoiLA: Volatile recycling in the Lesser Antilles. *Eos* **100**, (2019).

49. H. Kopp, W. Weinzierl, A. Becel, P. Charvis, M. Evain, E. R. Flueh, A. Gailler, A. Galve, A. Hirn, A. Kandilarov, D. Klaeschen, M. Laigle, C. Papenberg, L. Planert, E. Roux, Deep structure of the central Lesser Antilles Island Arc: Relevance for the formation of continental crust. *Earth Planet. Sci. Lett.* **304**, 121–134 (2011).

50. M. Paulatto, M. Laigle, A. Galve, P. Charvis, M. Sapin, G. Bayrakci, M. Evain, H. Kopp, Dehydration of subducting slow-spread oceanic lithosphere in the Lesser Antilles. *Nat. Commun.* **8**, 15980 (2017).

51. I. Jackson, U. H. Faul, Grainsize-sensitive viscoelastic relaxation in olivine: Towards a robust laboratory-based model for seismological application. *Phys. Earth Planet. Inter.* **183**, 151–163 (2010).

52. C. Havlin, B. K. Holtzman, E. Hopper, Inference of thermodynamic state in the asthenosphere from anelastic properties, with applications to North American upper mantle. *Phys. Earth Planet. Inter.* **314**, 106639 (2021).

53. U. H. Faul, I. Jackson, Transient creep and strain energy dissipation: An experimental perspective. *Annu. Rev. Earth Planet. Sci.* **43**, 541–569 (2015).

54. Y. Takei, Effects of partial melting on seismic velocity and attenuation: A new insight from experiments. *Annu. Rev. Earth Planet. Sci.* **45**, 447–470 (2017).

55. G. A. Abers, P. E. van Keken, B. R. Hacker, The cold and relatively dry nature of mantle forearcs in subduction zones. *Nat. Geosci.* **10**, 333–337 (2017).

56. J. Corbeau, O. Gonzalez, N. Feuillet, A. Lejeune, F. R. Fontaine, V. Clouard, J. Saurel; OVSM Team, A significant increase in interplate seismicity near major historical earthquakes offshore martinique (FWI). *Bull. Seismol. Soc. Am.* **111**, 3118–3135 (2021).

57. F. Halpaap, S. Rondenay, A. Perrin, S. Goes, L. Ottemöller, H. Austrheim, R. Shaw, T. Eeken, Earthquakes track subduction fluids from slab source to mantle wedge sink. *Sci. Adv.* **5**, eaav7369 (2019).

58. D. Arcay, Dynamics of interplate domain in subduction zones: Influence of rheological parameters and subducting plate age. *Solid Earth* **3**, 467–488 (2012).

59. H. Yamauchi, Y. Takei, Polycrystal anelasticity at near-solidus temperatures. *J. Geophys. Res. Solid Earth* **121**, 7790–7820 (2016).

60. G. Wadge, Comparison of volcanic production rates and subduction rates in the Lesser Antilles and Central America. *Geology* **12**, 555–558 (1984).

61. M. Bohm, C. Haberland, G. Asch, Imaging fluid-related subduction processes beneath Central Java (Indonesia) using seismic attenuation tomography. *Tectonophysics.* **590**, 175–188 (2013).

62. T. Chen, R. W. Clayton, Seismic attenuation structure in central Mexico: Image of a focused high-attenuation zone in the mantle wedge. *J. Geophys. Res. Solid Earth* **114**, B07304 (2009).

63. E. Hopper, H. A. Ford, K. M. Fischer, V. Lekic, M. J. Fouch, The lithosphere–asthenosphere boundary and the tectonic and magmatic history of the northwestern United States. *Earth Planet. Sci. Lett.* **402**, 69–81 (2014).

64. N. G. Cerpa, I. Wada, C. R. Wilson, Effects of fluid influx, fluid viscosity, and fluid density on fluid migration in the mantle wedge and their implications for hydrous melting. *Geosphere* **15**, 1–23 (2019).

65. N. Feuillet, I. Manighetti, P. Tapponnier, E. Jacques, Arc parallel extension and localization of volcanic complexes in Guadeloupe, Lesser Antilles. *J. Geophys. Res. Solid Earth* **107**, ETG 3-1–ETG 3-29 (2002).

66. Institut De Physique Du Globe De Paris (IPGP), Ecole Et Observatoire Des Sciences De La Terre De Strasbourg (EOST), *GEOSCOPE, French Global Network of broad band seismic stations* (IPGP, Université de Paris, 1982); http://geoscope.ipgp.fr/networks/detail/G/.

67. Institut De Physique Du Globe De Paris (IPGP), *GNSS, seismic broadband and strong motion permanent networks in West Indies* (IPGP, Université de Paris, 2008); http://volobsis.ipgp.fr/networks/detail/WI/.

68. S. S. Wei, D. A. Wiens, P-wave attenuation structure of the Lau back-arc basin and implications for mantle wedge processes. *Earth Planet. Sci. Lett.* **502**, 187–199 (2018).

69. K. Aki, P. G. Richards, *Quantitative Seismology* (University Science Books, 2002).

70. J. G. Anderson, S. E. Hough, A model for the shape of the fourier amplitude spectrum of acceleration at high frequencies. *Bull. Seismol. Soc. Am.* **74**, 1969–1993 (1984).

71. M. Lindner, A. Rietbrock, L. Bie, S. Goes, J. Collier, C. Rychert, N. Harmon, S. P. Hicks, T. Henstock; VoiLA working group, Bayesian regional moment tensor from ocean bottom seismograms recorded in the Lesser Antilles: Implications for regional stress field. *Geophys. J. Int.* **233**, 1036–1054 (2022).

72. G. A. Prieto, R. L. Parker, F. L. Vernon III, A Fortran 90 library for multitaper spectrum analysis. *Comput. Geosci.* **35**, 1701–1710 (2009).

73. R. Madariaga, Dynamics of an expanding circular fault. *Bull. Seismol. Soc. Am.* **66**, 639–666 (1976).

74. I. Jackson, J. D. F. Gerald, U. H. Faul, B. H. Tan, Grain-size-sensitive seismic wave attenuation in polycrystalline olivine. *J. Geophys. Res. Solid Earth* **107**, ECV 5-1–ECV 5-16 (2002).

75. D. Eberhart-Phillips, M. Chadwick, S. Bannister, Three-dimensional attenuation structure of central and southern South Island, New Zealand, from local earthquakes. *J. Geophys. Res. Solid Earth* **113**, B05308 (2008).

76. A. Rietbrock, P wave attenuation structure in the fault area of the 1995 Kobe earthquake. *J. Geophys. Res. Solid Earth* **106**, 4141–4154 (2001).

77. S. P. Hicks, A. Rietbrock, I. M. Ryder, C.-S. Lee, M. Miller, Anatomy of a megathrust: The 2010 M8.8 Maule, Chile earthquake rupture zone imaged using seismic tomography. *Earth Planet. Sci. Lett.* **405**, 142–155 (2014).

78. F. Crameri, G. E. Shephard, P. J. Heron, The misuse of colour in science communication. *Nat. Commun.* **11**, 5444 (2020).

79. L. Krischer, T. Megies, R. Barsch, M. Beyreuther, T. Lecocq, C. Caudron, J. Wassermann, ObsPy: A bridge for seismology into the scientific Python ecosystem. *Comput. Sci. Discov.* **8**, 014003 (2015).

80. S. Goes, J. Armitage, N. Harmon, H. Smith, R. Huismans, Low seismic velocities below mid-ocean ridges: Attenuation versus melt retention. *J. Geophys. Res. Solid Earth* **117**, B12403 (2012).
